# Cystine rather than cysteine is the preferred substrate for β-elimination by cystathionine γ-lyase: implications for dietary methionine restriction

**DOI:** 10.1007/s11357-023-00788-4

**Published:** 2023-05-23

**Authors:** Thomas M. Jeitner, Juan A. Azcona, Gene P. Ables, Diana Cooke, Mark C. Horowitz, Pradeep Singh, James M. Kelly, Arthur J. L. Cooper

**Affiliations:** 1 Department of Radiology, Weill Cornell Medicine, 1300 York Avenue, New York, NY 10065 USA; 2 Department of Biochemistry and Molecular Biology, New York Medical College, Valhalla, NY 10595 USA; 3 Orentreich Foundation for the Advancement of Science, Inc, 855 Route 301, Cold Spring, NY 10516 USA; 4 Department of Orthopedics and Rehabilitation, Yale University School of Medicine, New Haven, CT 06510 USA; 5 Citigroup Biomedical Imaging Center, Weill Cornell Medicine, 516 East 72Nd St, New York, NY 10021 USA

**Keywords:** Cystine, Cysteine, Methionine restriction, Transsulfuration, Cystathionine beta-synthase, Cystathionine gamma-lyase

## Abstract

Dietary methionine restriction (MR) increases longevity by improving health. In experimental models, MR is accompanied by decreased cystathionine β-synthase activity and increased cystathionine γ-lyase activity. These enzymes are parts of the transsulfuration pathway which produces cysteine and 2-oxobutanoate. Thus, the decrease in cystathionine β-synthase activity is likely to account for the loss of tissue cysteine observed in MR animals. Despite this decrease in cysteine levels, these tissues exhibit increased H_2_S production which is thought to be generated by β-elimination of the thiol moiety of cysteine, as catalyzed by cystathionine β-synthase or cystathionine γ-lyase. Another possibility for this H_2_S production is the cystathionine γ-lyase-catalyzed β-elimination of cysteine persulfide from cystine, which upon reduction yields H_2_S and cysteine. Here, we demonstrate that MR increases cystathionine γ-lyase production and activities in the liver and kidneys, and that cystine is a superior substrate for cystathionine γ-lyase catalyzed β-elimination as compared to cysteine. Moreover, cystine and cystathionine exhibit comparable *K*_cat_/*K*_m_ values (6000 M^−1^ s^−1^) as substrates for cystathionine γ-lyase-catalyzed β-elimination. By contrast, cysteine inhibits cystathionine γ-lyase in a non-competitive manner (*K*_i_ ~ 0.5 mM), which limits its ability to function as a substrate for β-elimination by this enzyme. Cysteine inhibits the enzyme by reacting with its pyridoxal 5′-phosphate cofactor to form a thiazolidine and in so doing prevents further catalysis. These enzymological observations are consistent with the notion that during MR cystathionine γ-lyase is repurposed to catabolize cystine and thereby form cysteine persulfide, which upon reduction produces cysteine.

**Supplementary information:**

The online version contains supplementary material available at 10.1007/s11357-023-00788-4.

## Introduction

Reducing calories or the content of certain amino acids in diets extends life in a healthful manner. These beneficial effects result from delaying the onset of various fatal diseases [1–3]. Thus, elucidating the mechanisms by which diets increase longevity is likely to yield fundamental insights into how tissues age and become diseased. Of the dietary modifications that improve lifespan, methionine restriction (MR) has received considerable attention. In this context, MR diets improve insulin sensitivity, reduce adiposity, and promote the hepatic metabolism of glucose and lipids [4–11].

The effects of MR are attributed, in part, to the increased production hydrogen sulfide (H_2_S) [12], as catalyzed by cystathionine γ-lyase (CGL) acting on either cysteine [13] or cysteine disulfide (cystine) [14]. Throughout this communication, cystine and cysteine refer to the L enantiomers of these amino acids: L,L-cystine and L-cysteine. The increase in H_2_S production due to MR parallels an increase in CGL transcription [15–18]. Most publications describing a role of CGL in MR favor the utilization of cysteine by CGL to produce H_2_S by the following β-elimination reaction:

1 $$\mathrm{Cysteine }+ {\mathrm{H}}_{2}\mathrm{O}\to \mathrm{ pyruvate }+ {\mathrm{H}}_{2}\mathrm{S }+ {{\mathrm{NH}}_{4}}^{+}$$

There is however little evidence for the occurrence of this reaction in biological systems. By contrast, CGL could produce cysteine persulfide from cystine by a β-elimination reaction [14, 19, 20]:

2 $$\begin{array}{lc}\mathrm{Cystine }+ {\mathrm{H}}_{2}\mathrm{O }\\ \to \text{cysteine persulfide }+\mathrm{ pyruvate }+ {{\mathrm{NH}}_{4}}^{+}\end{array}$$

Cysteine persulfide could in turn act as a source of H_2_S following thiol (RSH)-disulfide interchange:

3 $$\text{Cysteine persulfide} + \mathrm{ RSH } \to \text{cysteine-SR }+ {\mathrm{H}}_{2}\mathrm{S}$$

**Fig. 1:**
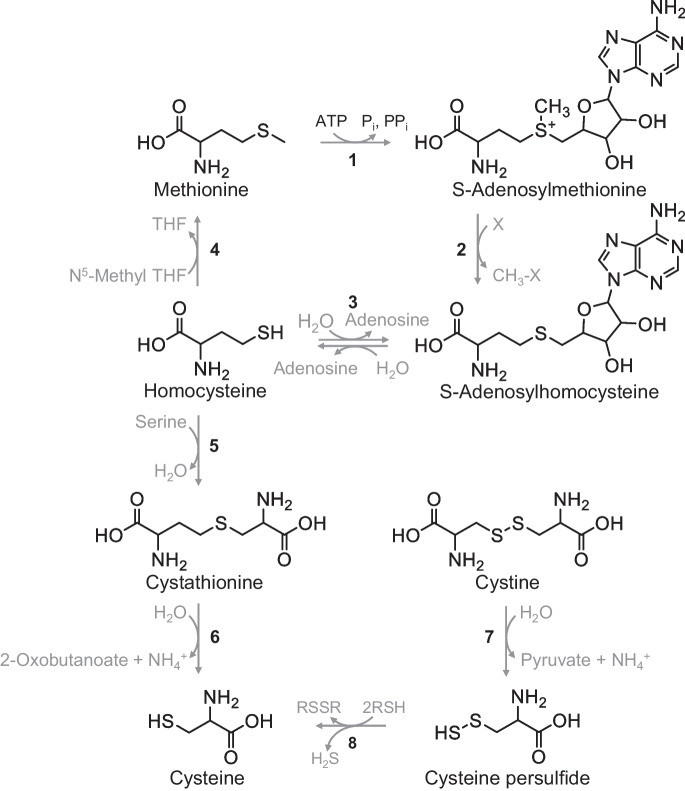
Cysteine biosynthesis via methionine or cystine. Reactions 1 to 4 describe the *methionine cycle* whereby methionine is converted to S-adenosylmethionine by methionine adenosyltransferase (1). S-Adenosylmethionine is in turn converted to S-adenosylhomocysteine by methyltransferases (2) that donate the methyl group of S-adenosylmethionine to various acceptors. The adenosine moiety of S-adenosylhomocysteine is then cleaved from homocysteine by adenosylhomocysteinase (3). Finally, methylation of homocysteine by methionine synthase yields methionine to complete the cycle (4). Folate metabolism provides the methyl donor for the latter reaction in the form of N.^5^-methyltetrahydrofolate. Homocysteine can also be methylated to methionine by betaine-homocysteine methyltransferase using glycine betaine as the methyl donor (not shown). Reactions 5 to 6 describe *transsulfuration* in which cystathionine β-synthase condenses homocysteine and serine to form cystathionine (5) which CGL then cleaves into cysteine and 2-oxobutanoate (6). CGL *also cleaves* cystine to yield cysteine persulfide (7) which undergoes reduction (e.g., thiols: RSH) to produce H_2_S and the corresponding disulfides (RSSR) (8)

These reactions are also depicted in Fig. 1. We favor the indirect mechanism for H_2_S production by CGL acting on cystine, as outlined by reactions 2 and 3, for chemical and biological reasons.

The chemical reasons rely on the fact that cystine is a much better homolog of cystathionine than is cysteine for binding at the active site of CGL (*c.f.* the structure of cystathionine with the structure of cystine in Fig. 1), and the nature of the leaving groups for reactions 1 and 2. The latter point is an important consideration because the cleavage of carbon–sulfur bonds by lyases depends on the ability of the putative leaving group to accept electrons. In the case of the first reaction, the leaving group is the thiol moiety of cysteine (-SH), whereas in the second reaction, the leaving group is the persulfide moiety of cystine (-SSCH_2_CH(NH_2_)CO_2_H). Of these compounds, cysteine persulfide is the better leaving group, which argues in favor of H_2_S production beginning with the actions of CGL on cystine.

The biological reason for favoring cystine as a substrate for CGL during MR is that cystathionine β-synthase activity is significantly attenuated due to decreases in the amounts of this enzyme and its substrate homocysteine, as supplied from the methionine cycle [15, 21]. This results in decrements in cystathionine production, as reflected in the low plasma concentrations of this metabolite [15, 22] and low cellular levels of cysteine (Fig. 1, reactions 1–4) [23]. The low levels of cystathionine and cysteine under these conditions may favor utilization of cystine by CGL as a substrate. In this study, we sought to distinguish between the two hypotheses for H_2_S production, namely that H_2_S is produced through β-elimination reactions with either cysteine or cystine as catalyzed by CGL.

## Materials

All materials were purchased from Sigma-Aldrich unless stated otherwise. Cystine and homocystine were dissolved in 1 N HCl as 50 and 80 mg mL^−1^ solutions, respectively, and then neutralized for use in the assays. Homocysteine-cysteine disulfide was produced as described by Büdy et al. [24] and the product confirmed by ^1^H-NMR and mass spectrometry.

## Methods

### Animal care

C57BL/6 J male mice were purchased from the Jackson Laboratories (Bar Harbor, ME), group housed (5 mice/cage) in the animal facility maintained by the Yale Animal Resource Center. Food and water were provided *ad libitum*. The diet ingredients and feeding protocol have been described previously [25]. Briefly, upon arrival, the mice were acclimatized for 1 week and fed Teklad Global Rodent Diet (18% protein, vegetarian, 2018S, East Millstone, NJ). Afterwards, the mice were weight matched and separated into either a control group fed a standard diet (control fed (CF), 0.84% methionine w/w) or into a group fed an MR diet (0.12% methionine w/w). In both diets, the total calorie intact was as follows: 14% kcal protein, 76% kcal carbohydrate, and 10% kcal fat (Research Diets, New Brunswick, NJ) for 8 weeks. The other components of these diets are listed in Cooke et al. [25]; neither diet includes cysteine and the low methionine diet contains additional glutamate to offset the decrease in methionine. Mice were 6- to 8-week-old at the initiation of the experiments and 16-week-old at termination. All experiments were approved by the Yale Institutional Animal Care and Use Committee. Animal experiments were also approved by the Institutional Animal Care and Use Committee of the Orentreich Foundation for the Advancement of Science, Inc. (Permit Number: 0511 MB), as previously described (29).

Liver and kidneys were removed, flash frozen in liquid nitrogen, and stored at − 80 °C until analyzed. Bone marrow and adipose tissue were also removed (next two sections) and stored at − 80 °C until analyzed for CGL activity. For analysis of CGL activities, slices of frozen tissues were shaved from the frozen bulk using a razor blade into a volume of ice-cold 100 mM potassium phosphate buffer (pH 7.2) and 1% p Halt™ Protease Inhibitor Cocktail (Thermo Fisher Scientific) equal to 4 times tissue mass. The shavings and any remaining tissue were then minced further on ice using the blade and then transferred to glass Dounce homogenizers for further grinding on ice consisting of 5 pestle strokes. These materials were then placed in 1.5-mL tubes and centrifuged at 1000 × g for 15 min at 4 °C. The supernatant was used for the determination of protein content and CGL assay. Protein content was determined by the BCA method (Thermo Fisher Scientific). The supernatants were snap frozen in liquid nitrogen and stored at − 80 °C until analyzed.

### Isolation of bone marrow and adipose tissue from CF and MR mice

Tibias and femurs were isolated and cleaned of soft tissue. The epiphyses were removed, and the bones placed in an Eppendorf tube, which contained a conical insert such that the end of the bones remained just above the bottom of the Eppendorf tube. The bones were centrifuged at 3800 × g for 30 s. The conical insert containing the bones was discarded and the bone marrow pellet snap frozen by emersion in liquid nitrogen. These tissues were homogenized as described above for the liver and kidney samples. Subcutaneous white and brown adipose tissue were isolated, cleaned of connective tissue, placed in 1.5-mL tubes, and snap frozen by emersion in liquid nitrogen.

### Purification of rat liver CGL

Rat liver CGL was partially purified from rat liver by the method of Hargrove and Wichman [26]. Further chromatography using mono Q or mono S columns did not increase the purity of enzyme. Therefore, a chromatographic fraction containing CGL purified to 53% purity was used for these studies. Enzyme purity and protein content were determined as described in the Supplementary Fig. 1.

### CGL activity measurements

In addition to catalyzing a γ-elimination reaction with L,L-cystathionine, CGL also catalyzes a γ-elimination of 2-oxobutanoate (α-ketobutyrate) from L-homoserine [Eq. 4], and this reaction is commonly used to monitor CGL activity [27].

4 $$\text{Homoserine}\to \text{2-oxobutanoate }+ {{\mathrm{NH}}_{4}}^{+}$$

For this assay, the reaction mixture contains 50 mM L-homoserine, 100 mM potassium phosphate buffer (pH 7.2), and the enzyme source in a final volume of 50 µL [28]. The reaction begins with the addition of homoserine to the mixture for incubation at 37 °C for varying periods depending on activities present in the tissues. For liver and kidney samples, we incubated the reaction mixtures for either 5 or 10 min; by contrast, the reaction mixtures containing fatty tissues were incubated for 18 h. The reaction is terminated by the addition of 20 µL 5 mM 2,4-dinitrophenylhydrazine in 2 M HCl and incubated for a further 10 min, after which 130 µL of 1 M NaOH is added and the absorbance of 2-oxobutanoate 2,4-dinitrophenylhydrazone at 430 nm is determined using an extinction coefficient of 16,100 M^−1^ cm^−1^. A similar method is used when pyruvate is the eliminated product except that absorbance of pyruvate 2,4-dinitrophenylhydrazone at 430 nm is determined using an extinction coefficient of 17,700 M^−1^ cm^−1^. Absorbances are measured within 2 min of the addition of NaOH. The extinction coefficients were calculated by first measuring the actual amounts of 2-oxobutanoate or pyruvate present in a solution prepared at a concentration of 100 µM in 100 mM potassium phosphate buffer (pH 7.2). This was done by measuring the extent to which NADH was oxidized by this solution in the presence of rabbit lactate dehydrogenase (LDH). Graded amounts of the calibrated solutions were then reacted with 2,4-dinitrophenylhydrazine in 2 M HCl (as described above) and the extinction coefficients derived as the slopes of the linear plots of absorbance at 430 nm against the µM amounts of 2-oxobutanoate or pyruvate. An extinction coefficient of 6220 M^−1^ cm^−1^ for NADH was used for these calculations.

### ^1^NMR and mass spectrometry

The reaction between 10 mM pyridoxal 5′-phosphate (PLP) and 80 mM cysteine was studied by NMR after dissolving PLP and cysteine to the aforementioned concentrations in 100 mM potassium phosphate (pH 7.2) buffer in D_2_O. ^1^H spectra were acquired on a 500 MHz Bruker Avance III spectrometer. Chemical shifts δ are expressed in parts per million, with the residual solvent resonance as an internal standard (D_2_O, 4.79 ppm). The first NMR spectrum was recorded after 6 min of mixing. The disappearance of aldehyde peak at 10.43 ppm and appearance at 5.98 ppm in the mixture suggest the formation of 1,3-thiazolidine derivative (Fig. 7B and supplementary Fig. 2).

Furthermore, we monitored the reaction between PLP and cysteine by Waters Acquity Ultra Performance Liquid Chromatography (UPLC) using a Phenomenox Kinetex® Evo C18 100 Å column (2.1 mm × 50 mm; 1.7 mm), a 5–95% gradient of acetonitrile in water with 0.1% formic acid as mobile phase, and a flow rate of 0.4 mL/min. A UPLC chromatogram demonstrated the formation of a 1,3-thiazolidine derivative from a mixture of PLP (10 mM) and cysteine (80 mM) in phosphate buffer (pH 7.2). The 1,3-thiazolidine derivative peak appeared at 0.67 min; [M + H]^+^ exact mass: 351.0; and found: 350.9 (Supplementary Fig. 3).

### UV–visible spectral studies of the reaction of PLP and cysteine

The reactions were performed with mixtures consisting of 100 mM potassium phosphate buffer (pH 7.2), 50 µM PLP, and 50 to 400 µM concentrations of cysteine at 22 °C. One reaction mixture contained PLP whereas the other cysteine. Each reaction began with the rapid combination of 0.8 mL volumes of PLP and cysteine-containing solutions 5 s prior to the collection of the first scan from 290 to 450 nm. Additional scans were acquired at minute intervals thereafter. All scans were collected at a rate of 24,000 nm/min.

### UV–visible spectral studies of human CGL reacting with cysteine

Spectra of CGL 100 µg or 2.25 nmol human recombinant CGL (Cayman Chemicals, 95% pure) in a 400 µL volume consisting of 100 mM potassium phosphate (pH 7.2) and either 0 or 100 µM cysteine at 22 °C were collected from 290 to 450 nm at a rate of 24,000 nm/min. Spectra were collected every 2 min and smoothed using Prism software. The raw plots are given in Supplementary Data Fig. 4.

## Results

### CGL is variably expressed in murine tissues and MR increases the hepatic and renal amounts and activities of CGL

**Fig. 2:**
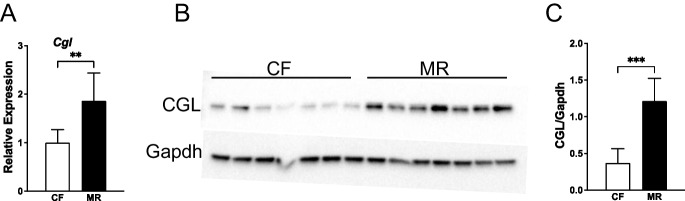
Hepatic CGL gene and protein expression are upregulated in MR mice. **A** Gene expression was determined by quantitative real-time PCR (qPCR) using TAQMAN primers normalized to β-actin, as described in the “Methods” section (**P < 0.01, *n* = 7–8/group). **B** Protein expression was determined by Western blot as described in the “Methods” section. Bands represent proteins from liver tissue samples of control-fed (CF) or methionine-restricted (MR) mice. **C** Band intensities (**P < 0.001, *n* = 7/group) were quantified as described in the “Methods” section

**Fig. 3:**
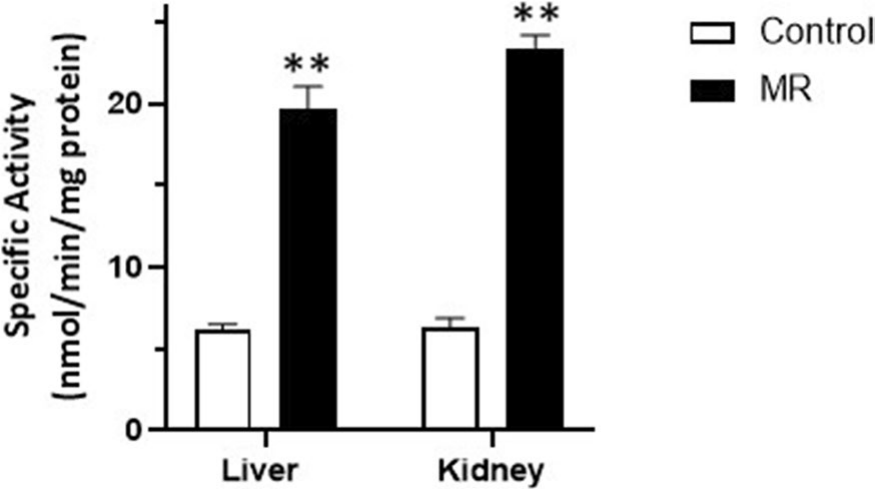
Specific activity of hepatic and renal CGL. Shown are hepatic CGL specific activities from 11 mice fed either normal or MR diet and the renal CGL activities of 5 mice fed similarly. The data are depicted as the averages ± SEM and the significant differences at *P* < 0.01 as **. These differences were determined using the *t*-test

MR increases CGL message in inguinal white adipose tissue [29] and liver [18, 29]. In the case of the liver, increases in CGL activities accompany the increases in mRNA message due to MR [15–18, 29]. However, as shown in Table 1, not all tissues express CGL and those that express CGL mRNA do not necessarily produce the enzyme in an active form. Therefore, we sought to confirm the increases in hepatic CGL expression and activities due to MR and to measure CGL activities in various fatty tissues, liver, and the kidneys. Dietary MR results in significant increases in the amounts of hepatic CGL protein, mRNA, and specific activities present in C57BL/6 J mice (Figs. 2 and 3). CGL protein and specific activities increase approximately threefold in MR as compared to mice fed normal chow. The increases in hepatic CGL specific activities are comparable to the increases in renal CGL activities in MR mice (Fig. 3). Despite the reported elevation of CGL mRNA in inguinal white adipose tissues [16], the baseline specific activity of CGL in bone marrow or visceral, subcutaneous, or brown adipose is very low (< 0.1% of that exhibited by liver—data not shown) and difficult to measure. We were only able to measure CGL activity after overnight incubation at 37 °C. Even so, the variability of these measurements precluded an accurate assessment of the effect on MR on bone marrow and adipose tissue.

**Table 1:**
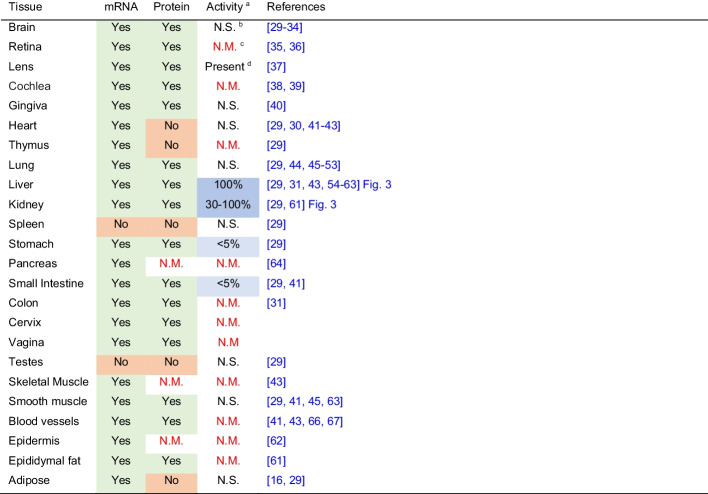
CGL mRNA, protein, and enzyme activities in select mammalian tissues [29–34] [35, 36] [37] [38, 39] [40] [29, 30, 41–43] [29] [29, 44–53] [29, 31, 43, 48, 54–63] [29, 61] [29] [29] [64] [29, 41] [31] [29] [43] [29, 41, 45, 65] [41, 43, 66, 67] [62] [61] [16, 29]

^a^Determined by the catalytic conversion of a substrate and not as inferred from H_2_S production by tissues

^b^Not significant

^c^Not measured

^d^Based on the available data, it is not possible to estimate the activities relative to the liver

### CGL preferentially uses cystine rather than cysteine as a substrate

**Fig. 4:**
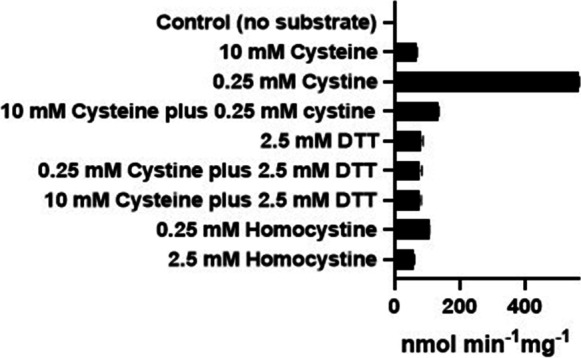
CGL preferentially uses cystine as a substrate. Here, the catalysis of elimination reactions with various amino acids by rat liver CGL is shown. The data for this study was obtained as described in the “Methods” section using 360 ng rat liver CGL (8.26 pmol) in a reaction volume of 50 µL and is shown as the average ± SD of 3 replicates

To determine whether CGL preferentially acts on cystine or cysteine, we measured the efficiency of CGL to catalyze a β-elimination with these substrates to produce pyruvate as per reactions 1 and 2. Although some activity was noted with 10 mM cysteine, the amount of pyruvate formed is considerably less than the amount formed with 0.25 mM cystine as the substrate (Fig. 4). In these experiments, we were constrained by the upper limit of cystine’s solubility at 0.25 mM. Nonetheless, we tested cysteine at a concentration of 10 mM to ensure the possibility of observing pyruvate production due to the actions of CGL. Even though CGL acting on 10 mM cysteine produced a small amount of pyruvate, 10 mM cysteine impeded the production of pyruvate due to the action of CGL on 0.25 mM cystine. Cysteine-cystine disulfide exchange cannot be an explanation for this pyruvate production because there will be no net change in cystine concentration. Another explanation, and one explored below, is that cysteine acts as an inhibitor of CGL. In considering the earlier reports of H_2_S generated by CGL acting on cysteine [13], we posit that the solutions of cysteine used in these earlier studies may have been oxidized to cystine as originally suggested by Cavallini et al. [14]. Cysteine oxidizes to cystine in the presence of oxygen and alkaline solutions [68]. To test this possibility, we reduced 0.25 mM cystine to 0.5 mM cysteine using an excess of DTT and then measured pyruvate formation as catalyzed by CGL. The reduction of cystine by DTT resulted in pyruvate production by CGL at levels comparable to the production by 10 mM cysteine and a dramatic decrease in production compared to 0.25 mM cystine alone. The fact that 0.5 and 10 mM cysteine produce comparable amounts of pyruvate is explicable given that cysteine inhibits CGL at higher concentrations (see below and [69]). Cysteine and cysteine plus DTT generate equivalent amounts of pyruvate indicating that cysteine is a substrate for this enzyme, although a very poor one as compared to cystine. To assess the substrate specificity of CGL-catalyzed elimination reactions, we tested homocystine as a substrate for γ-elimination by this enzyme:

5 $$\begin{array}{lc}\text{Homocystine} + \mathrm{H}_{2}\mathrm{O}\\ \to \mathrm{homocysteine persulfide} + \mathrm{2-oxobutanoate} + \mathrm{NH}^{+}_{4}\end{array}$$

Homocystine was found to be a much poorer substrate than is cystine even at a concentration ten times greater than that of cystine.

### Kinetic parameters of cystine as a CGL substrate

**Fig. 5:**
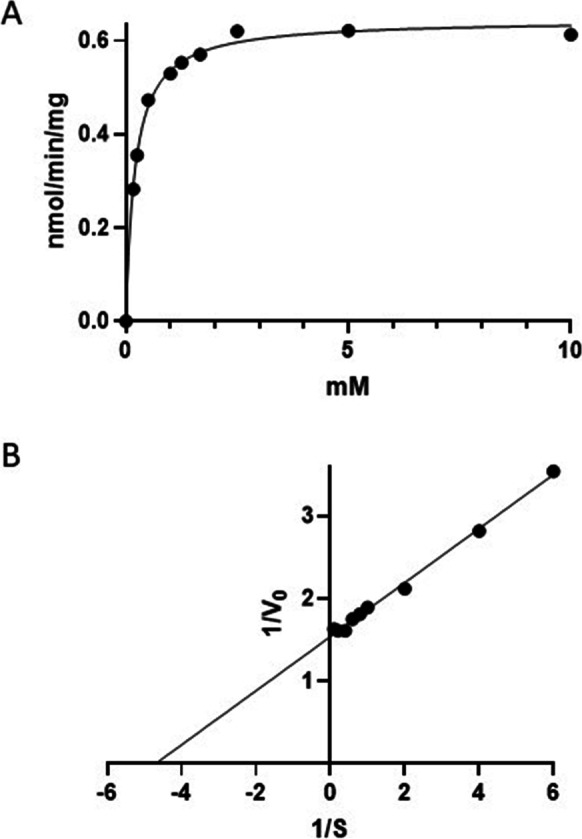
Cystine is a CGL substrate. Here, the kinetics of CGL-catalyzed 2-oxobutanoate production using cystine as a substrate is shown. The data for this study was obtained as described in the “Methods” section using 360 ng rat liver CGL (8.26 pmol) in a reaction volume of 50 µL. **A** The data as function of cystine concentrations; **B** the double reciprocal plot of the same data. The data are depicted as the average ± SD of triplicate determinations, while the plots were fitted using Prism software. In both cases, the plots have *R*.^2^ values of 1.00

Having demonstrated that cystine is a preferred substrate for β-elimination by CGL, we then determined the kinetic parameters of this reaction (Fig. 5) to compare its substrate behavior with that of cystathionine and other CGL substrates (Table 2). Cystine exhibits saturable kinetic behavior as a substrate for β-elimination by CGL which was used to determine the parameters depicted in Fig. 5B. We also derived the same parameters for cystathionine, homoserine, homocysteine-cysteine disulfide, and homocystine and ordered these reactions according to their catalytic efficiency, *i.e.*, their *V*_max_/*K*_m_ values (Table 2). As shown in this table, cystine and cystathionine exhibit equivalent catalytic efficiencies as CGL substrates. However, this equivalence reflects different substrate behaviors for these compounds; specifically, the *K*_m_ of CGL for cystine is *tenfold less* than the *K*_m_ of CGL for cystathionine, while the *V*_max_ for CGL acting on cystine is *tenfold more* than for CGL acting on cystine. The combination of these parameters produces the equivalent *V*_max_/*K*_m_ values. These parameters are explicable in terms of the physical chemistry of these compounds. At neutral pH, cystine precipitates at concentrations of 0.25 mM and above. A *K*_m_ of 0.22 mM therefore ensures an enzymatic means for removing cystine from the cytosol before it can accumulate to dangerous concentrations. The lower *V*_max_ for the reaction of CGL acting on cystine as compared to the *V*_max_ for the reaction with cystathionine (*i.e.*, 0.66 *versus* 6.78 nmol min^−1^ mg^−1^) can be attributed to the leaving group in these reactions, namely pyruvate and 2-oxobutanoate, respectively; 2-oxobutanoate is expected to be a better leaving group as per the rules associated with leaving groups [70].

**Table 2:** Kinetic characteristics of the reaction of CGL acting on various amino acids. The data for this study was obtained as described in Fig. 5 for the compounds indicated below

Reactions	*K*_m_ (mM)	*V*_max_ (nmol min^−1^ mg^−1^)	*K*_cat_ (s^−1^)	*K*_cat_/*K*_m_ (M^−1^ s^−1^)
Cystine → cysteine persulfide + pyruvate + NH_4_^+^	0.22 ± 0.00 (3)^§^	0.66 ± 0.01 (3)	1.33	6093
Cystathionine → cysteine + 2-oxobutanoate + NH_4_^+^	2.28 ± 0.07 (3)	6.78 ± 0.41 (3)	13.7	6008
Homoserine → 2-oxobutanoate + NH_4_^+^	12.4 ± 0.23 (3)	11.6 ± 0.55 (3)	23.4	1897
Homocysteine-cysteine → homocysteine persulfide + pyruvate + NH_4_^+^	82.0 ± 3.14 (3)	1.73 ± 0.94 (3)	3.49	42.6
Homocystine → homocysteine persulfide + 2-oxobutanoate + NH_4_^+^	60.7 ± 1.48 (3)	0.45 ± 0.64 (3)	0.92	15.1

^§^Mean ± SE (*n*)

2-Oxobutanoate is similarly released by the action of CGL on homoserine and accounts for its use as a convenient CGL substrate (reaction 4) [27]: The catalytic efficiency of CGL acting on homoserine of 1897 M^−1^ s^−1^ reflects a superior *K*_cat_ of 23.4 s^−1^ as compared to cystathionine which has a *K*_cat_ of 13.7 s^−1^. Even so, CGL preferentially binds and catalyzes reactions with sulfur-containing amino acids, which is reflected in the higher *K*_m_ of CGL for homoserine *versus* the *K*_m_ of CGL for cystathionine: 12.4 *versus* 2.28 mM, which lowers the *V*_max_/*K*_m_ for homoserine despite its high *K*_cat_.

CGL exhibits significant selectivity with respect to the disulfides accessing its substrate binding site. As indicated in Fig. 4, homocystine is a poor substrate for β-elimination as catalyzed by CGL. This property results from a high *K*_m_ exhibited by CGL for homocystine of 60.7 mM, which is 275 times greater than its *K*_m_ for cystine and reduces the* V*_max_/*K*_m_ of CGL acting on homocystine to less than 0.25% of the value obtained with cystine. Homocystine differs from cystine by the presence of additional methylene groups on either side of the disulfide moiety (CO_2_H(NH_2_)CH_2_CH_2_CHSSCH_2_CH_2_CH(NH_2_)CO_2_H *vs*. CO_2_H(NH_2_)CH_2_CHSSCH_2_CH(NH_2_)CO_2_H). Homocysteine-cysteine disulfide ﻿(CO_2_H(NH_2_)CH_2_CH_2_CHSSCH_2_CH(NH_2_)CO_2_H), which lacks one of these additional methylene groups, allows CGL greater access to the carbon–sulfur bond in the cysteinyl residue, as indicated by the higher *K*_cat_ of 3.49 s^−1^ for the CGL acting of homocysteine-cysteine disulfide and as compared to the *K*_cat_ of 0.92 s^−1^ for CGL acting on homocystine. The difference in these *K*_cat_ values is sufficient to overcome the large difference in *K*_m_ values for affinities of homocysteine-cysteine disulfide and homocystine for CGL of 82.0 and 60.7, respectively, and to produce a *V*_max_/*K*_m_ for CGL acting on homocysteine-cysteine disulfide and CGL that is 2.8-fold greater than that exhibited by CGL acting on homocystine. Even so, the *V*_max_/*K*_m_ value obtained with homocysteine-cysteine disulfide as a substrate for CGL is still only less than 0.7% of the value obtained for CGL acting on cystine. Thus, based on catalytic efficiency, the rank order for disulfides acting as CGL substrates is cystine < < homocysteine-cysteine disulfide < homocystine.

### Cysteine is an inhibitor of CGL

**Fig. 6:**
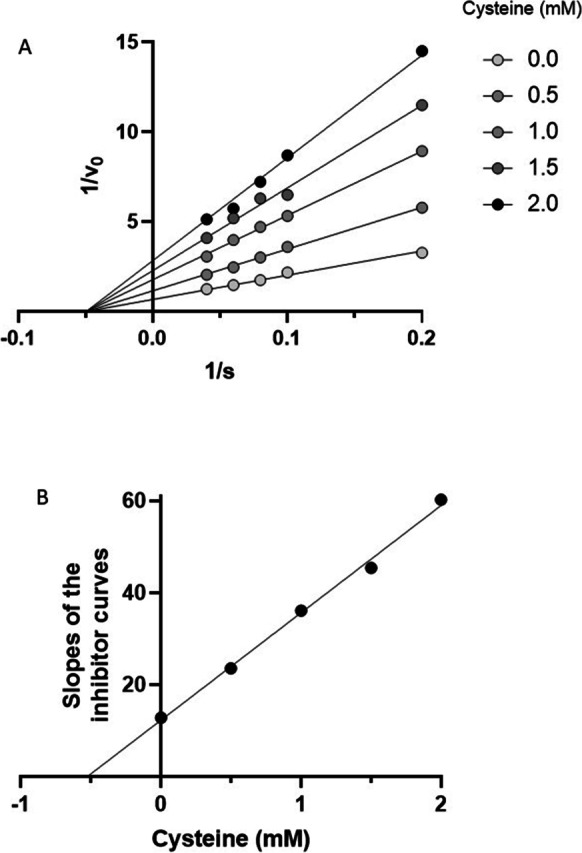
Cysteine is an inhibitor of CGL. **A** Here, cysteine is shown to be a non-competitive inhibitor of CGL with respect to conversion of homoserine to 2-oxobutanoate. **B** The Dixon plot indicates that the *K*_i_ for this inhibition is 0.52 mM. The data for this study were obtained as described in the “Methods” section using 228 ng rat liver CGL (6.42 pmol) in a reaction volume of 50 µL. The data are depicted as the means of up to 9 determinations. For the sake of clarity, the SD values are not depicted but can be found in Supplementary Table 1. All plots were fitted using Prism software and have *R*^2^ values of 1.00

Two observations suggest that cysteine acts as a CGL inhibitor: the decrement in pyruvate production by CGL acting on cystine due to inclusion of cysteine in the reaction mixture (Fig. 4) and the formation of thiazolidine adduct of PLP following its reaction with cysteine [71, 72]. The formation of such an adduct would inhibit the catalytic cycle of PLP-containing enzymes such as CGL. Given these observations, we investigated the effect of cysteine on the β-elimination of H_2_O from homoserine by CGL (Fig. 6). The data depicted in Fig. 6A is consistent with cysteine acting as a non-competitive inhibitor of CGL with respect to homoserine. A Dixon replot of the data yields a *K*_i_ of 0.52 mM for this inhibition (Fig. 6B).

**Fig. 7:**
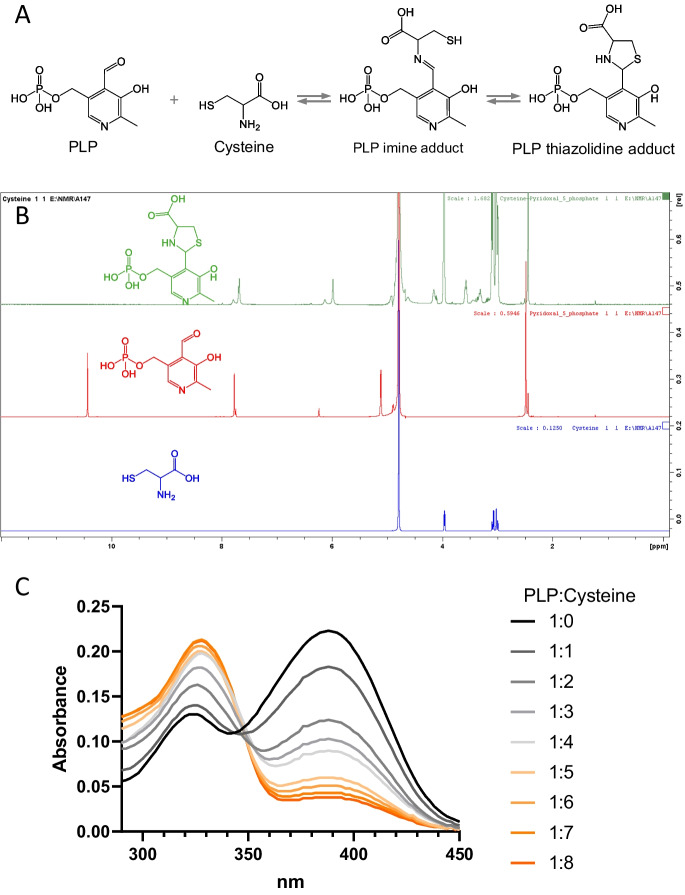
Reaction of cysteine with PLP. **A** Scheme for the reaction of protein bound PLP with cysteine. **B** Thiazolidine formation from the reaction PLP with cysteine. **C** Changes in the absorbance of 50 µM PLP reacting with 50, 100, 150, 200, 250, 300, 350, and 400 µM cysteine. Each trace represents the mean of at least 7 scans. The mean ± SD (*n*) of these scans is presented in the Supplementary Table 2

Cysteine binds to both free and enzyme-bound PLP to form a thiazolidine as shown in Fig. 7A [71, 72]. This reaction could therefore account for the non-competitive inhibition of CGL by cysteine. The thiazolidine in Fig. 7A was described as a *cis* (2S,4R) or a *trans* (2R,4R) isomer of 2-(3-hydroxy-5-phosphonooxymethyl-2-methyl-4-pyridyl)-1,3-thiazolidine-4-carboxylic acid [73]. For the sake of simplicity, we use Terzuoli et al.’s convention of referring to this mixture of diastereoisomers as a thiazolidine.

The formation of the thiazolidine shown in Fig. 7A was demonstrated in earlier studies by ^1^HNMR under acidic conditions [73], and here, we confirmed its production at near-neutral pH by ^1^HNMR and mass spectrometry (Fig. 7B and Supplementary Fig. 3). We also investigated this reaction using UV–visible spectrophotometry. PLP exhibits a spectrum with maxima at 322 and 388 nm (Fig. 7C). The addition of cysteine to PLP solutions causes the absorbance at 322 nm to increase at the expense of absorbance at 388 nm.

**Fig. 8:**
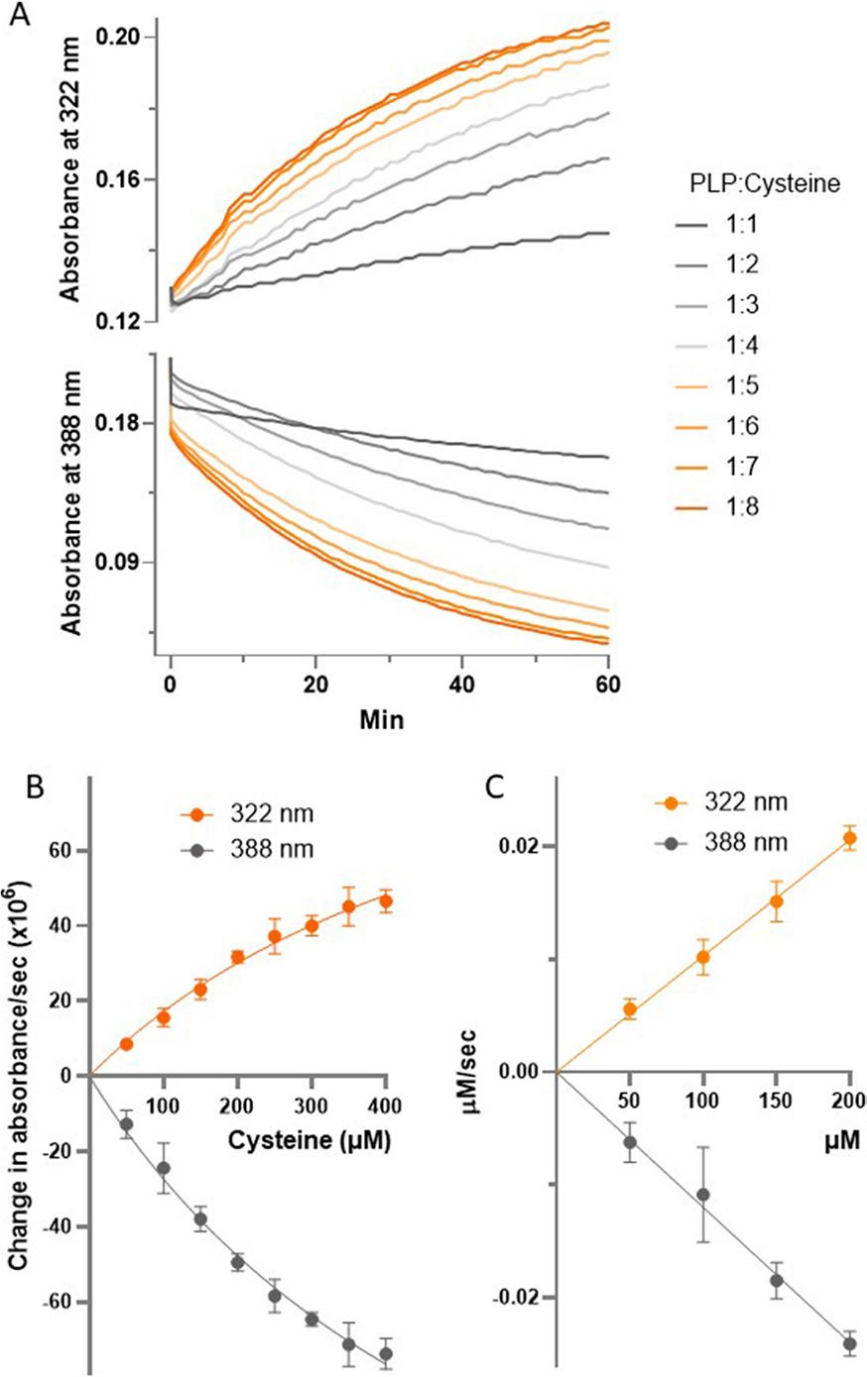
Determination of the rate constant for the reaction of cysteine and PLP. **A** The changes in absorbance at 322 and 388 nm due to reaction of 50 µM PLP and 50, 100, 150, 200, 250, 300, 350, and 400 µM cysteine (depicted as PLP:cysteine ratios: 1:1, 1:2, 1:3, 1:4, 1:5, 1:6, 1:7, and 1:8, respectively) are shown as a function of time. The data were extracted from scans such as those depicted in Fig. 7 and includes data taken from scans acquired first at 5 s and then at successive minute intervals following the initial mixing of PLP and cysteine. Because these traces would overlap on a contiguous *Y* axis, the *Y* axis was spilt into two regions to enable viewing of changes at 322 and 388 nm independently of each other. Each trace represents the mean of at least 4 data points. The mean ± SD (*n*) of these absorbances is presented in the Supplementary Table 3. The initial velocities of the data shown in **A** are depicted changes in optical density and concentrations over time in **B** and **C**, respectively

In Fig. 8A, the changes due to 60 min of reaction with PLP and cysteine are shown. These changes in absorbance were derived from scans initiated at 5-s and 1-min intervals following the addition of PLP and cysteine. Consequently, we determined the absorbance changes at 322 and 388 nm as a function of time (Fig. 8B). Mixing effects were evident at the 5-s and 1-min data points for some traces (e.g., a suppression of absorbances at 322 nm below the absorbance of PLP at this wavelength). Even so, it is possible to measure rate changes by ignoring the mixing effects and determining rates of change between 1 and 10 min for the progress curves shown in Fig. 8A. These rate changes are presented in Fig. 8B and demonstrate reciprocal changes at 322 and 388 nm that reflect the consumption of PLP to form the PLP thiazolidine adduct. In agreement with this interpretation, the rate changes at 322 and 388 nm are inversely correlated (*R*^2^ = 1.00, Supplementary Fig. 4). As shown in Fig. 8B, the rate changes are linear with respect to cysteine concentrations up 200 µM, consistent with a first order reaction between PLP and cysteine. To estimate the rate constants for the loss of PLP and the formation of the thiazolidine due to the reaction of PLP and cysteine, we determined extinction coefficients 1530 and 2060 M^−1^ cm^−1^ for the absorbance changes at 322 and 388 nm, respectively (Supplementary Data Fig. 5). Using these coefficients, we produced the plots shown in Fig. 8C and derived rate constants of 1.03 ± 0.01 × 10^−4^ and 1.20 ± 0.03 × 10^−4^ s^−1^ for the formation of the thiazolidine and loss of PLP, respectively. Given the concentrations of CGL and cysteine used in the experiments depicted in Fig. 5, namely 165 nM enzyme versus 0.5 to 2.0 mM cysteine, a fold difference of 0.3 to 1.2 × 10^4^, the rate constant for the reaction of PLP and cysteine of 10^−4^ s^−1^ is sufficient to account for the inhibition of CGL by cysteine reacting with its PLP cofactor. The addition of cystine to PLP produces no changes in PLP spectrum.

**Fig. 9:**
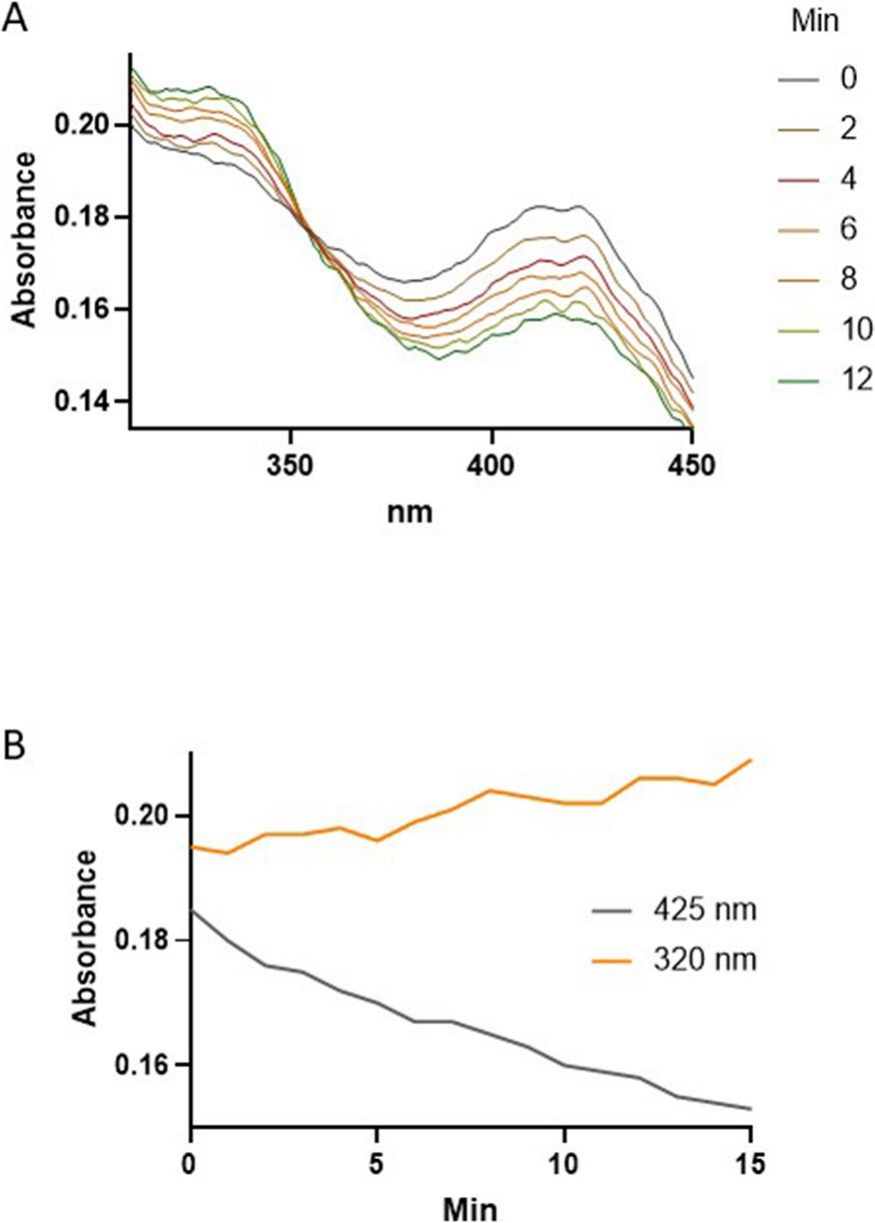
Cysteine induces spectral changes in CGL indicative of a reaction with PLP. Spectra of 100 µg or 2.25 nmol of human recombinant CGL in 400 µL (5.617 µM) prior to and following the addition of cysteine at a final concentration of 100 µM are shown (**A**). The kinetic of the changes in absorbance at 320 and 450 nm is depicted (**B**). The reaction was carried out in 100 mM potassium phosphate buffer (pH 7.2) and at 22 °C. Spectra were collected every 1 min and smoothed using Prism software. The raw plots are given in Supplementary Data Fig. 4

The absorbance due to PLP dominates the UV–vis spectrum of CGL (Fig. 9). The PLP aldimine formed from a lysyl residue in the CGL active site absorbs at 427 nm [69, 74]. Therefore, we tested whether the addition of cysteine to recombinant human CGL produces changes in the enzyme’s spectrum comparable to those depicted in Fig. 7B for the reaction of PLP and cysteine. The addition of cysteine at a final concentration of 100 µM to a solution containing recombinant human CGL induces spectral changes comparable to those observed with PLP and cysteine, though at 320 nm and 427 nm rather than at 322 and 388 nm. These spectral changes are in agreement with those reported by Yamagata et al. for yeast CGL incubated with cysteine [69]. The changes in absorbance at 320 and 427 nm (Fig. 9B) are linearly correlated (*R*^2^ = 0.87) for 15 min of the reaction, suggesting the loss of enzyme-bound PLP at the expense of forming a PLP adduct. Taken together, these observations indicate that cysteine, at concentrations higher than 0.5 mM, inhibits this enzyme by forming a thiazolidine with its PLP cofactor.

## Discussion

Here, we report that rat liver CGL preferentially catalyzes a β-elimination reaction with cystine rather than with cysteine. In the case of cystine, CGL-catalyzed β-elimination produces cysteine persulfide, a putative source of cysteine and H_2_S. The catalytic efficiency of this reaction compares well with the efficiency of the reaction with cystathionine as the substrate for β-elimination by CGL. By contrast, high concentrations of cysteine inhibit CGL activity in a non-competitive manner by forming a thiazolidine with its PLP cofactor.

The liver and kidneys contain the greatest amounts of CGL activities (Table 1) [29] and MR increases the transcription of hepatic and renal CGL [15–18]. Because CGL transcripts are not always translated (Table 1), we confirmed that the increase in transcription resulted in robust increases in hepatic and renal CGL protein and specific activities. However, under the conditions of our experiments, little CGL activity is evident in bone marrow or visceral, subcutaneous, or brown adipose. The lack of CGL activity in most tissues (Table 1) [29] suggests the possibility that transsulfuration and possibly cystine degradation may be confined to select tissues, primarily the liver and kidneys. Cells export homocysteine and cystine. Therefore, extra-hepatic and renal tissues can forgo transsulfuration and cystine degradation by exporting homocysteine and cystine. These observations imply that MR, at least in terms of its effects on transsulfuration, mainly affects the kidneys and liver. What these effects are remains open to question, because H_2_S production is often preceded by the generation of sulfane sulfur species, such as cysteine persulfide as catalyzed by CGL (reaction 2) or 3-mercaptopyryvate sulfurtransferase [75, 76]. These species could therefore account for some of the effects of MR attributed to H_2_S [77].

Elucidating the contributions of cystine and cysteine to the effects of MR requires some understanding of the extent to which these sulfur-containing amino acids serve as substrates for CGL. At sub-mM concentrations, cysteine acts as a substrate, albeit a poor one, for β-elimination by *S. cerevisiae* CGL to produce H_2_S. Yamagata et al. reported that the activity produced with cysteine as a substrate for β-elimination by *S. cerevisiae* CGL produces a tenth of the activities obtained using cystine as a substrate, in agreement with the findings reported here [69]. Similarly, the *K*_cat_/*K*_m_ value for cysteine acting as a substrate for β-elimination by recombinant human CGL is a very small fraction of the value obtained using cystathionine as the substrate: 190 vs 4200 M^−1^ s^−1^, respectively [74]. In addition to describing the substrate behavior of cysteine for yeast CGL, Yamagata et al. demonstrated that mM amounts of cysteine inhibit this enzyme [69], a finding substantiated by other researchers working with the CGL from other simple organisms [78–80]. Despite this discovery, the mechanism by which cysteine inhibits CGL had not hitherto been elucidated. Determining this mechanism and the *K*_i_ for the inhibition are important for considerations of cysteine’s role in metabolism. Cysteine inhibits CGL by forming a thiazolidine with the catalytic PLP aldimine in the active site of the enzyme. This mechanism has now been verified by UV–vis spectrometry and ^1^H-NMR using the CGL of various species and PLP (this work and [69, 71, 72, 81–83]). Moreover, the formation of a thiazolidine accounts for the inhibition of other PLP-containing enzymes by cysteine [81, 84]. We confirmed the results of earlier studies as the basis for testing the hypothesis that this reaction might account for the non-competitive inhibition of CGL by cysteine. Specifically, we sought to determine whether the rate of the reaction is sufficiently fast to account for this inhibition. Buell and Hanson had calculated a rate constant for the reaction of PLP and cysteine, assuming the reaction is second order, of 1.7 × 10^−1^ M^−1^ s^−1^ at pH 7.0 [71]. We, however, determined that the reaction is first order, with a rate constant of 10^−4^ s^−1^ that is sufficiently rapid to account for the inhibition of CGL by cysteine.

Cysteine inhibits mammalian CGL with a *K*_i_ of 520 µM, a concentration that lies above the cysteine concentrations normally found in cells: ~ 300 µM [85–87]. Lui et al. hypothesize that supraphysiological concentrations of cysteine act as a feedback inhibitor of PLP-containing enzymes [81]. This implies that as intracellular cysteine concentrations fall below the *K*_i_ values for the inhibition of these enzymes, cysteine or metabolites thereof (e.g., pyruvate, ammonia, and H_2_S as per Yang et al. [82]) dissociate from the thiazolidine to reform the PLP cofactor. The dissociation rates of cysteine or its metabolites from either the CGL bound or free thiazolidine are not known. Studies of the thiazolidine adduct report on the appearance of PLP or pyridoxine after days rather than a rate per se [71, 73, 83]. In the same vein, Terzuoli et al. calculated an equilibrium constant for the dissociation of the thiazolidine of 36.8 M^−1^ at 37 °C for a PLP:cysteine stoichiometry of 3:4 [73]. Terzuoli’s calculation indicates a propensity for dissociation, especially if cysteine is being consumed, but this is not the case in cells. As noted above, cellular cysteine levels are maintained at 10^−4^ M concentrations which would not favor the dissociation of thiazolidine formed at the active site in PLP enzymes. Moreover, dissociation of the thiazolidine would be even less favored at the higher cellular cysteine levels that inhibit CGL. Taken together, these observations support an argument that the formation of a thiazolidine by cysteine in PLP enzymes is an essentially irreversible act. The cysteine-PLP thiazolidine adduct exists in plasma [88]. Thus, formation of the cysteine-PLP thiazolidine may lead to expulsion of this entity from the active site yielding the enzyme in an inactive apo form. The enzyme could then be reconstituted with PLP [74]. The occurrence of these events and their regulation of PLP enzymes in a feedback manner remain to be verified. Nevertheless, given the slow dissociation of the thiazolidine and the slow association of PLP with apo CGL thus far reported, it is more likely that the activities catalyzed by CGL are regulated transcriptionally rather than through thiazolidine formation [74]. In the case of MR, this appears to be the case as the number of CGL transcripts increases following a lowering of dietary methionine intake [15–18].

The beneficial effects of MR have been attributed to the actions of CGL that produce H_2_S via a β-elimination from cysteine (Eq. 1) [12]. However, although our data and those of Yamagata et al. [69] show that the amount of H_2_S generated by this mechanism is likely to be small. In this regard, cystine is a much better source for H_2_S production than is cysteine. As a substrate for β-elimination by mammalian CGL, cystine is at least as good a substrate as cystathionine yielding *K*_cat_/*K*_m_ values of 6093 and 6008 M^−1^ s^−1^, respectively. By contrast, the *K*_cat_/*K*_m_ value for β-elimination of cysteine by CGL (190 M^−1^ s^−1^) is 30-fold lower that our *K*_cat_/*K*_m_ value for CGL-catalyzed β-elimination of cystathionine and 20 fold lower than the *K*_cat_/*K*_m_ value obtained by Zhu et al. for the reaction catalyzed by human CGL [74]. Thus, CGL, by acting on cystine, has a greater potential to contribute to the cellular H_2_S pool, by way of cysteine persulfide production, than by acting on cysteine.

The discussion has thus far focused on the idea that H_2_S contributes to the effects of MR in a significant manner, but in the case of the events resulting from the actions of cystathionine γ-lyase, cysteine rather than H_2_S may be the more important product derived from the reduction of cysteine persulfide (Fig. 1, reaction 8). Thus, the production of cysteine persulfide (as catalyzed by CGL acting on cystine) could be a source of much needed cysteine during MR when the possibility of obtaining cysteine via transsulfuration is significantly attenuated. MR attenuates cystathionine β-synthase activity and thereby the flux of homocysteine though transsulfuration [15, 21]. Consequently, plasma homocysteine levels rise while plasma cystathionine and tissue cysteine levels fall [15, 21, 89–91]. Despite these changes, three apparently paradoxical events occur during MR: the amounts and activities of the other major transsulfuration enzyme, CGL, increase in the kidneys and liver (this study and [15–18]) as do the amounts of H_2_S and plasma glutathione (which is derived from the liver) [12, 92]. The production of glutathione or H_2_S requires cysteine which is not readily available via transsulfuration [75, 93]. Banerjee and colleagues estimate that under normal conditions, approximately half of the cysteine utilized for GSH production in the liver is derived from transsulfuration [23]. CGL and cystathionine β-synthase catalyze the β-elimination of H_2_S from cysteine [74, 93], whereas 3-mercaptopyruvate sulfurtransferase catalyzes the formation of sulfane sulfur (which could theoretically be reduced to H_2_S) from cysteine-derived 3-mercaptopyruvate [75]. In the absence of transsulfuration, the generation of H_2_S through these processes will decrease [15, 21]. Thus, during MR, the production of cysteine resulting from the actions of CGL on cystine could act as alternate source of cysteine for glutathione and protein synthesis. The same reaction also produces H_2_S and likely more H_2_S than does CGL acting on cysteine (discussed above). Autophagy also increases during MR [94–96]. One of the products generated by autophagy is cystine [97, 98]. Therefore, we posit that MR causes increases in both CGL and cystine. Elimination reactions with amino disulfides as catalyzed by CGL are restricted to two very structurally similar compounds—cystine and cystathionine—and not the larger analogs, homocystine, or homocysteine-cysteine disulfide. In the absence of cystathionine, CGL catabolizes cystine to cysteine persulfide, which upon reduction yields cysteine for glutathione and protein synthesis (as well as H_2_S). In other words, MR causes CGL to be repurposed to catabolize what is thought to be a cellular waste product, cystine, to generate a source of metabolically useful cysteine, namely cysteine persulfide.

### Supplementary Information

Below is the link to the electronic supplementary material.

Supplementary file1 (DOCX 26.1 KB)

Supplementary file2 (DOCX 89.8 KB)

Supplementary file3 (DOCX 89.5 KB)

Supplementary Figure 1: Determination of the rat liver CGL protein concentrations. Chromatographic fractions of CGL and graded amounts of BSA were separated by SDS PAGE, stained with Coomassie Blue G250 and then quantified using ImageJ software. The stained gels for three separate experiments are depicted below. The protein content of the CGL fractions was estimated each gel based on the linear regression of pixels versus BSA amounts as determined using the Prism software. In each case, the R2 values for the linear regression is 1.00. (JPG 44 KB)

Supplementary Figure 2: Assignments of the protons in thiazolidine formed by the reaction of cysteine and PLP. (JPG 33 KB)

Supplementary Figure 3: Mass spectrometry of the thiazolidine formed by the reaction of cysteine and PLP. (JPG 61 KB)

Supplementary Figure 4: Correlation between the absorbance changes at 322 and 388 nm for the reaction of PLP and cysteine. The rate changes at 388 nm are shown as a function of the rate changes at 322 nm for cysteine and PLP concentrations depicted in Figure 8. The data is taken from Figure 8B and represents mean ± SD values. (JPG 28 KB)

Supplementary Figure 5: Determination of the Extinction Coefficients for PLP-thiazolidine and PLP. Shown are the absorbance changes at 322 and 388 nm due to graded amounts of PLP thiazolidine and PLP, respectively. PLP thiazolidine was produced by reacting 1 mM PLP with 6 mM cystine in 100 mM potassium phosphate buffer (pH 7.2) for 15 min at 22°C, which the mixture was placed on ice. The optical densities of PLP thiazolidine concentrations at 322 nm was subtracted from the optical density for the equivalent concentrations of PLP at 322 nm. The data represents the mean values of 3 determinations that varied by less than 2% of each other. Linear regression using Prism software yielded the extinction coefficients of 1,530 and 2,060 M-1.cm-1 for the absorbance changes at 322 and 388 nm. In both cases the data was fitted to linear expressions with R2 values of 1.0. (JPG 24 KB)

Supplementary Figure 6: Cysteine induces spectral changes in CGL indicative of a reaction with PLP. Shown are the spectra of the reaction of human recombinant CGL and cysteine at final concentrations of 5.62 and 100 μM, respectively. The reaction was carried out in 100 mM potassium phosphate buffer (pH 7.2) and at 22°C and a volume of 400 μL. Spectra were collected at a rate of 24,000 nm/min and at every min of the reaction. (JPG 32 KB)

Supplementary Figure 7: Correlation between the absorbance changes at 322 and 388 nm for the reaction of CGL and cysteine The rate changes at 425 nm are shown as a function of the rate changes at 320 nm for cysteine and PLP concentrations depicted in Figure 9. (JPG 27 KB)

## Data Availability

The authors confirm that the data supporting the findings of this study are available within the article and/or its supplementary materials.
