# Supplementary material for: Cystine rather than cysteine is the preferred substrate for β-elimination by cystathionine γ-lyase: implications for dietary methionine restriction
Source: GeroScience. 2023 May 23;46(4):3617–34. doi: 10.1007/s11357-023-00788-4 (PMC11229439; doi:10.1007/s11357-023-00788-4)
Supplement: Supplementary file 1 — Supplementary file1 (DOCX 26.1 KB) [file 11357_2023_788_MOESM1_ESM.docx]

**Supplementary Table 1: Michaelis-Menten Analysis of Inhibition of CGL by Cysteine**

Shown are data for the double reciprocal plot depicted in Fig 6, *i.e.*, (nmol/min/mg)^-1^ *versus* substrate concentration^-1^.

|  | Cysteine(mM) | | | | | | | | | | | | | | | |
| --- | --- | --- | --- | --- | --- | --- | --- | --- | --- | --- | --- | --- | --- | --- | --- | --- |
| 1/Homoserine (mM) | 0 | 0.5 | | | 1 | | | | 1.5 | | | | 2 | | | |
| 0.04 | 1.24 ± 0.04 (9) § | 2.04 ± 0.07 (3) | | | 3.06 ± 0.21 (4) | | | | 4.09 ± 0.04 (5) | | | | 5.13 ± 0.25 (5) | | | |
| 0.06 | 1.47 ± 0.14 (9) | 2.45 ± 0.14 (3) | | | 3.98 ± 0.35 (4) | | | | 5.19 ± 0.12 (5) | | | | 5.73 ± 0.53 (5) | | | |
| 0.08 | 1.74 ± 0.12 (9) | 3.01 ± 0.21 (3) | | | 4.70 ± 0.36 (4) | | | | 6.29 ± 0.08 (5) | | | | 7.22 ± 0.82 (5) | | | |
| 0.10 | 2.17 ± 0.29 (9) | 3.59 ± 0.04 (3) | | | 5.32 ± 0.31 (4) | | | | 6.49 ± 0.69 (5) | | | | 8.69 ± 0.48 (5) | | | |
| 0.20 | 3.27 ± 0.06 (9) | 5.78 ± 0.42 (3) | | | 8.93 ± 0.45 (4) | | | | 11.5 ± 2.01 (5) | | | | 14.5 ± 3.07 (5) | | | |
| § Mean ± SE (n) |  |  |  |  | |  |  |  | |  |  |  | |  |  |  |
