# Supplementary material for: Cystine rather than cysteine is the preferred substrate for β-elimination by cystathionine γ-lyase: implications for dietary methionine restriction
Source: GeroScience. 2023 May 23;46(4):3617–34. doi: 10.1007/s11357-023-00788-4 (PMC11229439; doi:10.1007/s11357-023-00788-4)
Supplement: Supplementary file 2 — Supplementary file2 (DOCX 89.8 KB) [file 11357_2023_788_MOESM2_ESM.docx]

**Supplementary Table 2: Changes in the absorbance of 50 μM PLP reacting with 50, 100, 150, 200, 250, 300, 350, and 400 μM cysteine as depicted in Fig 7**

| **Mean Values** | | | | | | | | | |
| --- | --- | --- | --- | --- | --- | --- | --- | --- | --- |
| PLP (uM) | 50 | 50 | 50 | 50 | 50 | 50 | 50 | 50 | 50 |
| Cys (uM) |  | 50 | 100 | 150 | 200 | 250 | 300 | 350 | 400 |
| nm |  |  |  |  |  |  |  |  |  |
| 450 | 0.011 | 0.007 | 0.004 | 0.004 | 0.004 | 0.002 | 0.002 | 0.003 | 0.004 |
| 448 | 0.013 | 0.008 | 0.005 | 0.004 | 0.005 | 0.003 | 0.003 | 0.003 | 0.002 |
| 446 | 0.015 | 0.011 | 0.007 | 0.007 | 0.006 | 0.006 | 0.005 | 0.007 | 0.005 |
| 444 | 0.017 | 0.012 | 0.007 | 0.006 | 0.006 | 0.004 | 0.003 | 0.005 | 0.003 |
| 442 | 0.020 | 0.014 | 0.009 | 0.008 | 0.008 | 0.004 | 0.004 | 0.005 | 0.004 |
| 440 | 0.024 | 0.017 | 0.011 | 0.010 | 0.009 | 0.006 | 0.005 | 0.006 | 0.005 |
| 438 | 0.028 | 0.021 | 0.013 | 0.012 | 0.011 | 0.007 | 0.006 | 0.007 | 0.005 |
| 436 | 0.034 | 0.025 | 0.016 | 0.014 | 0.013 | 0.008 | 0.007 | 0.008 | 0.006 |
| 434 | 0.039 | 0.030 | 0.019 | 0.017 | 0.015 | 0.010 | 0.008 | 0.009 | 0.007 |
| 432 | 0.046 | 0.035 | 0.023 | 0.020 | 0.018 | 0.012 | 0.010 | 0.010 | 0.008 |
| 430 | 0.053 | 0.041 | 0.027 | 0.023 | 0.021 | 0.014 | 0.012 | 0.011 | 0.009 |
| 428 | 0.062 | 0.048 | 0.032 | 0.028 | 0.026 | 0.018 | 0.015 | 0.015 | 0.012 |
| 426 | 0.071 | 0.056 | 0.037 | 0.031 | 0.028 | 0.018 | 0.016 | 0.015 | 0.013 |
| 424 | 0.081 | 0.064 | 0.043 | 0.036 | 0.032 | 0.021 | 0.018 | 0.017 | 0.015 |
| 422 | 0.092 | 0.073 | 0.049 | 0.041 | 0.036 | 0.024 | 0.021 | 0.019 | 0.016 |
| 420 | 0.103 | 0.082 | 0.055 | 0.046 | 0.041 | 0.027 | 0.024 | 0.021 | 0.018 |
| 418 | 0.114 | 0.092 | 0.062 | 0.051 | 0.045 | 0.030 | 0.026 | 0.023 | 0.020 |
| 416 | 0.125 | 0.101 | 0.068 | 0.057 | 0.050 | 0.033 | 0.029 | 0.025 | 0.022 |
| 414 | 0.136 | 0.110 | 0.074 | 0.062 | 0.054 | 0.036 | 0.031 | 0.027 | 0.024 |
| 412 | 0.147 | 0.120 | 0.080 | 0.067 | 0.059 | 0.039 | 0.034 | 0.029 | 0.026 |
| 410 | 0.158 | 0.128 | 0.086 | 0.072 | 0.063 | 0.042 | 0.036 | 0.031 | 0.027 |
| 408 | 0.169 | 0.137 | 0.093 | 0.077 | 0.068 | 0.045 | 0.039 | 0.033 | 0.029 |
| 406 | 0.178 | 0.145 | 0.098 | 0.081 | 0.072 | 0.048 | 0.041 | 0.035 | 0.031 |
| 404 | 0.187 | 0.153 | 0.103 | 0.086 | 0.075 | 0.050 | 0.043 | 0.037 | 0.032 |
| 402 | 0.195 | 0.160 | 0.108 | 0.090 | 0.079 | 0.053 | 0.045 | 0.038 | 0.034 |
| 400 | 0.202 | 0.166 | 0.111 | 0.093 | 0.081 | 0.054 | 0.046 | 0.039 | 0.035 |
| 398 | 0.209 | 0.171 | 0.115 | 0.096 | 0.084 | 0.056 | 0.048 | 0.041 | 0.036 |
| 396 | 0.214 | 0.175 | 0.118 | 0.098 | 0.086 | 0.057 | 0.048 | 0.041 | 0.036 |
| 394 | 0.218 | 0.179 | 0.121 | 0.100 | 0.088 | 0.059 | 0.050 | 0.043 | 0.038 |
| 392 | 0.221 | 0.181 | 0.122 | 0.101 | 0.089 | 0.059 | 0.051 | 0.043 | 0.038 |
| 390 | 0.222 | 0.182 | 0.123 | 0.102 | 0.089 | 0.060 | 0.051 | 0.043 | 0.038 |
| 388 | 0.223 | 0.183 | 0.124 | 0.103 | 0.090 | 0.060 | 0.051 | 0.043 | 0.038 |
| 386 | 0.222 | 0.182 | 0.123 | 0.102 | 0.089 | 0.060 | 0.051 | 0.043 | 0.038 |
| 384 | 0.221 | 0.181 | 0.123 | 0.102 | 0.089 | 0.060 | 0.051 | 0.043 | 0.038 |
| 382 | 0.218 | 0.179 | 0.121 | 0.100 | 0.088 | 0.059 | 0.050 | 0.043 | 0.038 |
| 380 | 0.215 | 0.176 | 0.119 | 0.099 | 0.086 | 0.058 | 0.049 | 0.042 | 0.037 |
| 378 | 0.211 | 0.173 | 0.117 | 0.097 | 0.086 | 0.057 | 0.049 | 0.042 | 0.037 |
| 376 | 0.206 | 0.171 | 0.117 | 0.097 | 0.086 | 0.057 | 0.049 | 0.042 | 0.037 |
| 374 | 0.200 | 0.164 | 0.111 | 0.093 | 0.081 | 0.055 | 0.047 | 0.040 | 0.035 |
| 372 | 0.194 | 0.159 | 0.108 | 0.091 | 0.080 | 0.054 | 0.046 | 0.040 | 0.035 |
| 370 | 0.188 | 0.154 | 0.105 | 0.088 | 0.077 | 0.053 | 0.045 | 0.039 | 0.035 |
| 368 | 0.181 | 0.149 | 0.101 | 0.086 | 0.075 | 0.052 | 0.045 | 0.039 | 0.035 |
| 366 | 0.174 | 0.143 | 0.099 | 0.083 | 0.074 | 0.051 | 0.045 | 0.039 | 0.035 |
| 364 | 0.167 | 0.137 | 0.095 | 0.081 | 0.073 | 0.051 | 0.045 | 0.040 | 0.036 |
| 362 | 0.160 | 0.132 | 0.093 | 0.081 | 0.073 | 0.053 | 0.047 | 0.043 | 0.039 |
| 360 | 0.152 | 0.127 | 0.091 | 0.080 | 0.074 | 0.055 | 0.049 | 0.046 | 0.042 |
| 358 | 0.146 | 0.122 | 0.090 | 0.081 | 0.077 | 0.059 | 0.054 | 0.051 | 0.048 |
| 356 | 0.139 | 0.118 | 0.090 | 0.084 | 0.081 | 0.065 | 0.061 | 0.059 | 0.055 |
| 354 | 0.131 | 0.114 | 0.092 | 0.088 | 0.088 | 0.073 | 0.070 | 0.069 | 0.066 |
| 352 | 0.126 | 0.111 | 0.095 | 0.095 | 0.097 | 0.083 | 0.081 | 0.081 | 0.079 |
| 350 | 0.120 | 0.110 | 0.099 | 0.102 | 0.107 | 0.095 | 0.094 | 0.095 | 0.093 |
| 348 | 0.116 | 0.109 | 0.104 | 0.111 | 0.120 | 0.109 | 0.110 | 0.111 | 0.110 |
| 346 | 0.112 | 0.108 | 0.110 | 0.121 | 0.131 | 0.124 | 0.125 | 0.128 | 0.126 |
| 344 | 0.110 | 0.109 | 0.116 | 0.130 | 0.144 | 0.137 | 0.140 | 0.143 | 0.142 |
| 342 | 0.109 | 0.111 | 0.123 | 0.140 | 0.155 | 0.151 | 0.154 | 0.159 | 0.158 |
| 340 | 0.109 | 0.114 | 0.130 | 0.149 | 0.165 | 0.162 | 0.166 | 0.171 | 0.170 |
| 338 | 0.111 | 0.117 | 0.136 | 0.157 | 0.174 | 0.173 | 0.177 | 0.183 | 0.182 |
| 336 | 0.113 | 0.121 | 0.142 | 0.164 | 0.182 | 0.181 | 0.187 | 0.193 | 0.192 |
| 334 | 0.117 | 0.126 | 0.148 | 0.170 | 0.188 | 0.188 | 0.194 | 0.201 | 0.200 |
| 332 | 0.121 | 0.131 | 0.154 | 0.176 | 0.193 | 0.194 | 0.200 | 0.206 | 0.205 |
| 330 | 0.124 | 0.135 | 0.158 | 0.180 | 0.196 | 0.198 | 0.204 | 0.211 | 0.210 |
| 328 | 0.128 | 0.138 | 0.161 | 0.182 | 0.198 | 0.200 | 0.206 | 0.213 | 0.212 |
| 326 | 0.130 | 0.140 | 0.163 | 0.182 | 0.197 | 0.200 | 0.206 | 0.212 | 0.211 |
| 324 | 0.130 | 0.140 | 0.162 | 0.181 | 0.193 | 0.197 | 0.204 | 0.210 | 0.209 |
| 322 | 0.130 | 0.139 | 0.160 | 0.177 | 0.188 | 0.192 | 0.199 | 0.204 | 0.204 |
| 320 | 0.128 | 0.137 | 0.156 | 0.172 | 0.182 | 0.186 | 0.192 | 0.198 | 0.197 |
| 318 | 0.124 | 0.133 | 0.152 | 0.166 | 0.175 | 0.179 | 0.186 | 0.190 | 0.190 |
| 316 | 0.120 | 0.127 | 0.145 | 0.159 | 0.166 | 0.172 | 0.177 | 0.182 | 0.179 |
| 314 | 0.115 | 0.123 | 0.140 | 0.152 | 0.159 | 0.163 | 0.169 | 0.174 | 0.173 |
| 312 | 0.109 | 0.116 | 0.132 | 0.144 | 0.150 | 0.155 | 0.162 | 0.166 | 0.165 |
| 310 | 0.103 | 0.110 | 0.127 | 0.138 | 0.144 | 0.149 | 0.155 | 0.159 | 0.159 |
| 308 | 0.096 | 0.103 | 0.121 | 0.130 | 0.135 | 0.140 | 0.147 | 0.151 | 0.152 |
| 306 | 0.089 | 0.097 | 0.115 | 0.125 | 0.130 | 0.137 | 0.143 | 0.148 | 0.147 |
| 304 | 0.083 | 0.091 | 0.110 | 0.120 | 0.124 | 0.133 | 0.139 | 0.144 | 0.143 |
| 302 | 0.077 | 0.086 | 0.106 | 0.116 | 0.120 | 0.130 | 0.136 | 0.141 | 0.141 |
| 300 | 0.071 | 0.081 | 0.102 | 0.112 | 0.115 | 0.126 | 0.133 | 0.138 | 0.138 |
| 298 | 0.065 | 0.077 | 0.099 | 0.109 | 0.112 | 0.123 | 0.131 | 0.136 | 0.135 |
| 296 | 0.062 | 0.073 | 0.096 | 0.105 | 0.108 | 0.120 | 0.128 | 0.133 | 0.133 |
| 294 | 0.059 | 0.070 | 0.094 | 0.103 | 0.104 | 0.118 | 0.126 | 0.131 | 0.131 |
| 292 | 0.057 | 0.069 | 0.092 | 0.100 | 0.100 | 0.116 | 0.124 | 0.129 | 0.129 |
| 290 | 0.056 | 0.068 | 0.092 | 0.099 | 0.098 | 0.114 | 0.122 | 0.127 | 0.128 |

| **SD Values** | | | | | | | | | |
| --- | --- | --- | --- | --- | --- | --- | --- | --- | --- |
| PLP (uM) | 50 | 50 | 50 | 50 | 50 | 50 | 50 | 50 | 50 |
| Cys (uM) |  | 50 | 100 | 150 | 200 | 250 | 300 | 350 | 400 |
| nm |  |  |  |  |  |  |  |  |  |
| 450 | 0.004 | 0.004 | 0.001 | 0.002 | 0.004 | 0.001 | 0.002 | 0.006 | 0.002 |
| 448 | 0.004 | 0.004 | 0.001 | 0.003 | 0.004 | 0.001 | 0.002 | 0.006 | 0.002 |
| 446 | 0.004 | 0.004 | 0.001 | 0.003 | 0.003 | 0.001 | 0.002 | 0.006 | 0.002 |
| 444 | 0.004 | 0.005 | 0.001 | 0.002 | 0.004 | 0.001 | 0.002 | 0.006 | 0.001 |
| 442 | 0.004 | 0.005 | 0.001 | 0.003 | 0.004 | 0.001 | 0.002 | 0.006 | 0.002 |
| 440 | 0.004 | 0.005 | 0.001 | 0.003 | 0.004 | 0.002 | 0.002 | 0.005 | 0.001 |
| 438 | 0.004 | 0.006 | 0.002 | 0.003 | 0.004 | 0.001 | 0.002 | 0.006 | 0.001 |
| 436 | 0.004 | 0.007 | 0.002 | 0.003 | 0.004 | 0.001 | 0.002 | 0.006 | 0.001 |
| 434 | 0.004 | 0.007 | 0.002 | 0.003 | 0.004 | 0.001 | 0.002 | 0.006 | 0.002 |
| 432 | 0.004 | 0.008 | 0.003 | 0.003 | 0.004 | 0.001 | 0.002 | 0.006 | 0.002 |
| 430 | 0.004 | 0.009 | 0.003 | 0.003 | 0.004 | 0.001 | 0.002 | 0.006 | 0.002 |
| 428 | 0.004 | 0.011 | 0.003 | 0.004 | 0.005 | 0.002 | 0.003 | 0.006 | 0.002 |
| 426 | 0.004 | 0.012 | 0.004 | 0.004 | 0.005 | 0.002 | 0.003 | 0.006 | 0.002 |
| 424 | 0.005 | 0.013 | 0.005 | 0.004 | 0.005 | 0.002 | 0.003 | 0.006 | 0.002 |
| 422 | 0.005 | 0.015 | 0.005 | 0.004 | 0.005 | 0.002 | 0.003 | 0.006 | 0.002 |
| 420 | 0.005 | 0.016 | 0.006 | 0.005 | 0.005 | 0.002 | 0.003 | 0.007 | 0.002 |
| 418 | 0.005 | 0.018 | 0.007 | 0.005 | 0.006 | 0.002 | 0.003 | 0.006 | 0.002 |
| 416 | 0.005 | 0.020 | 0.008 | 0.005 | 0.006 | 0.002 | 0.004 | 0.007 | 0.002 |
| 414 | 0.005 | 0.021 | 0.008 | 0.006 | 0.006 | 0.002 | 0.004 | 0.007 | 0.003 |
| 412 | 0.006 | 0.023 | 0.009 | 0.006 | 0.006 | 0.003 | 0.004 | 0.007 | 0.003 |
| 410 | 0.006 | 0.025 | 0.010 | 0.007 | 0.007 | 0.003 | 0.005 | 0.007 | 0.003 |
| 408 | 0.006 | 0.026 | 0.010 | 0.007 | 0.007 | 0.003 | 0.005 | 0.007 | 0.003 |
| 406 | 0.006 | 0.028 | 0.011 | 0.008 | 0.007 | 0.003 | 0.005 | 0.007 | 0.003 |
| 404 | 0.006 | 0.029 | 0.011 | 0.008 | 0.007 | 0.003 | 0.005 | 0.008 | 0.003 |
| 402 | 0.007 | 0.030 | 0.012 | 0.008 | 0.008 | 0.003 | 0.006 | 0.008 | 0.003 |
| 400 | 0.007 | 0.031 | 0.012 | 0.008 | 0.008 | 0.004 | 0.005 | 0.008 | 0.003 |
| 398 | 0.007 | 0.032 | 0.013 | 0.009 | 0.008 | 0.004 | 0.006 | 0.008 | 0.004 |
| 396 | 0.007 | 0.033 | 0.013 | 0.009 | 0.008 | 0.004 | 0.006 | 0.008 | 0.003 |
| 394 | 0.007 | 0.033 | 0.014 | 0.009 | 0.008 | 0.004 | 0.006 | 0.008 | 0.004 |
| 392 | 0.007 | 0.034 | 0.013 | 0.009 | 0.008 | 0.004 | 0.006 | 0.008 | 0.004 |
| 390 | 0.007 | 0.034 | 0.014 | 0.009 | 0.008 | 0.004 | 0.006 | 0.008 | 0.004 |
| 388 | 0.007 | 0.034 | 0.014 | 0.009 | 0.008 | 0.004 | 0.006 | 0.008 | 0.004 |
| 386 | 0.007 | 0.034 | 0.014 | 0.009 | 0.008 | 0.004 | 0.006 | 0.008 | 0.004 |
| 384 | 0.007 | 0.034 | 0.014 | 0.009 | 0.008 | 0.004 | 0.006 | 0.008 | 0.004 |
| 382 | 0.007 | 0.033 | 0.013 | 0.009 | 0.008 | 0.004 | 0.006 | 0.008 | 0.004 |
| 380 | 0.007 | 0.033 | 0.013 | 0.009 | 0.008 | 0.004 | 0.006 | 0.008 | 0.004 |
| 378 | 0.007 | 0.032 | 0.013 | 0.009 | 0.008 | 0.004 | 0.006 | 0.008 | 0.004 |
| 376 | 0.007 | 0.030 | 0.013 | 0.009 | 0.008 | 0.004 | 0.006 | 0.008 | 0.004 |
| 374 | 0.007 | 0.030 | 0.012 | 0.008 | 0.008 | 0.003 | 0.006 | 0.008 | 0.003 |
| 372 | 0.008 | 0.029 | 0.011 | 0.008 | 0.008 | 0.003 | 0.006 | 0.008 | 0.003 |
| 370 | 0.007 | 0.028 | 0.011 | 0.008 | 0.008 | 0.003 | 0.005 | 0.008 | 0.003 |
| 368 | 0.008 | 0.027 | 0.010 | 0.007 | 0.007 | 0.003 | 0.005 | 0.008 | 0.003 |
| 366 | 0.007 | 0.026 | 0.010 | 0.007 | 0.008 | 0.003 | 0.005 | 0.008 | 0.003 |
| 364 | 0.007 | 0.024 | 0.010 | 0.007 | 0.007 | 0.003 | 0.005 | 0.008 | 0.003 |
| 362 | 0.007 | 0.023 | 0.009 | 0.007 | 0.007 | 0.002 | 0.004 | 0.008 | 0.003 |
| 360 | 0.007 | 0.022 | 0.009 | 0.007 | 0.007 | 0.002 | 0.004 | 0.008 | 0.003 |
| 358 | 0.007 | 0.020 | 0.009 | 0.007 | 0.007 | 0.002 | 0.004 | 0.008 | 0.003 |
| 356 | 0.007 | 0.018 | 0.009 | 0.007 | 0.007 | 0.002 | 0.004 | 0.008 | 0.003 |
| 354 | 0.007 | 0.016 | 0.009 | 0.007 | 0.007 | 0.002 | 0.003 | 0.008 | 0.002 |
| 352 | 0.007 | 0.014 | 0.010 | 0.008 | 0.007 | 0.003 | 0.003 | 0.008 | 0.002 |
| 350 | 0.007 | 0.012 | 0.011 | 0.008 | 0.007 | 0.003 | 0.003 | 0.008 | 0.002 |
| 348 | 0.007 | 0.010 | 0.012 | 0.009 | 0.007 | 0.004 | 0.004 | 0.008 | 0.002 |
| 346 | 0.006 | 0.009 | 0.013 | 0.009 | 0.007 | 0.005 | 0.004 | 0.008 | 0.003 |
| 344 | 0.007 | 0.009 | 0.014 | 0.009 | 0.007 | 0.006 | 0.005 | 0.009 | 0.002 |
| 342 | 0.007 | 0.009 | 0.014 | 0.010 | 0.007 | 0.007 | 0.006 | 0.009 | 0.002 |
| 340 | 0.007 | 0.010 | 0.015 | 0.010 | 0.007 | 0.008 | 0.006 | 0.009 | 0.003 |
| 338 | 0.007 | 0.010 | 0.015 | 0.010 | 0.007 | 0.008 | 0.006 | 0.009 | 0.003 |
| 336 | 0.007 | 0.011 | 0.014 | 0.010 | 0.007 | 0.008 | 0.007 | 0.009 | 0.003 |
| 334 | 0.007 | 0.011 | 0.014 | 0.010 | 0.007 | 0.009 | 0.007 | 0.009 | 0.003 |
| 332 | 0.007 | 0.011 | 0.013 | 0.009 | 0.007 | 0.009 | 0.007 | 0.009 | 0.003 |
| 330 | 0.007 | 0.011 | 0.012 | 0.009 | 0.007 | 0.009 | 0.006 | 0.009 | 0.003 |
| 328 | 0.007 | 0.011 | 0.011 | 0.009 | 0.007 | 0.008 | 0.006 | 0.009 | 0.003 |
| 326 | 0.007 | 0.011 | 0.010 | 0.009 | 0.007 | 0.008 | 0.006 | 0.009 | 0.003 |
| 324 | 0.007 | 0.010 | 0.009 | 0.008 | 0.007 | 0.008 | 0.006 | 0.009 | 0.003 |
| 322 | 0.007 | 0.010 | 0.007 | 0.008 | 0.006 | 0.007 | 0.006 | 0.008 | 0.003 |
| 320 | 0.007 | 0.010 | 0.006 | 0.008 | 0.006 | 0.007 | 0.005 | 0.008 | 0.003 |
| 318 | 0.007 | 0.009 | 0.005 | 0.008 | 0.006 | 0.007 | 0.005 | 0.009 | 0.003 |
| 316 | 0.007 | 0.009 | 0.006 | 0.008 | 0.006 | 0.006 | 0.006 | 0.009 | 0.003 |
| 314 | 0.007 | 0.009 | 0.004 | 0.007 | 0.006 | 0.006 | 0.005 | 0.009 | 0.003 |
| 312 | 0.007 | 0.010 | 0.004 | 0.007 | 0.006 | 0.005 | 0.005 | 0.008 | 0.003 |
| 310 | 0.007 | 0.010 | 0.005 | 0.007 | 0.007 | 0.004 | 0.005 | 0.009 | 0.003 |
| 308 | 0.007 | 0.010 | 0.005 | 0.007 | 0.007 | 0.004 | 0.005 | 0.009 | 0.003 |
| 306 | 0.007 | 0.011 | 0.005 | 0.008 | 0.006 | 0.004 | 0.005 | 0.009 | 0.004 |
| 304 | 0.007 | 0.012 | 0.006 | 0.008 | 0.006 | 0.004 | 0.005 | 0.009 | 0.003 |
| 302 | 0.007 | 0.013 | 0.007 | 0.008 | 0.006 | 0.004 | 0.005 | 0.009 | 0.003 |
| 300 | 0.008 | 0.014 | 0.008 | 0.009 | 0.006 | 0.004 | 0.006 | 0.009 | 0.003 |
| 298 | 0.008 | 0.015 | 0.009 | 0.009 | 0.006 | 0.004 | 0.006 | 0.009 | 0.003 |
| 296 | 0.008 | 0.016 | 0.011 | 0.009 | 0.007 | 0.003 | 0.006 | 0.009 | 0.003 |
| 294 | 0.007 | 0.017 | 0.012 | 0.010 | 0.007 | 0.003 | 0.006 | 0.009 | 0.003 |
| 292 | 0.008 | 0.018 | 0.013 | 0.011 | 0.007 | 0.003 | 0.006 | 0.009 | 0.003 |
| 290 | 0.008 | 0.019 | 0.015 | 0.011 | 0.007 | 0.003 | 0.006 | 0.009 | 0.003 |

| **N Values** | | | | | | | | | |
| --- | --- | --- | --- | --- | --- | --- | --- | --- | --- |
| PLP (uM) | 50 | 50 | 50 | 50 | 50 | 50 | 50 | 50 | 50 |
| Cys (uM) |  | 50 | 100 | 150 | 200 | 250 | 300 | 350 | 400 |
| nm |  |  |  |  |  |  |  |  |  |
| 450 | 10 | 19 | 9 | 11 | 7 | 11 | 9 | 9 | 7 |
| 448 | 10 | 19 | 9 | 11 | 7 | 11 | 9 | 9 | 7 |
| 446 | 10 | 19 | 9 | 11 | 7 | 11 | 9 | 9 | 7 |
| 444 | 10 | 19 | 9 | 11 | 7 | 11 | 9 | 9 | 7 |
| 442 | 10 | 19 | 9 | 11 | 7 | 11 | 9 | 9 | 7 |
| 440 | 10 | 19 | 9 | 11 | 7 | 11 | 9 | 9 | 7 |
| 438 | 10 | 19 | 9 | 11 | 7 | 11 | 9 | 9 | 7 |
| 436 | 10 | 19 | 9 | 11 | 7 | 11 | 9 | 9 | 7 |
| 434 | 10 | 19 | 9 | 11 | 7 | 11 | 9 | 9 | 7 |
| 432 | 10 | 19 | 9 | 11 | 7 | 11 | 9 | 9 | 7 |
| 430 | 10 | 19 | 9 | 11 | 7 | 11 | 9 | 9 | 7 |
| 428 | 10 | 19 | 9 | 11 | 7 | 11 | 9 | 9 | 7 |
| 426 | 10 | 19 | 9 | 11 | 7 | 11 | 9 | 9 | 7 |
| 424 | 10 | 19 | 9 | 11 | 7 | 11 | 9 | 9 | 7 |
| 422 | 10 | 19 | 9 | 11 | 7 | 11 | 9 | 9 | 7 |
| 420 | 10 | 19 | 9 | 11 | 7 | 11 | 9 | 9 | 7 |
| 418 | 10 | 19 | 9 | 11 | 7 | 11 | 9 | 9 | 7 |
| 416 | 10 | 19 | 9 | 11 | 7 | 11 | 9 | 9 | 7 |
| 414 | 10 | 19 | 9 | 11 | 7 | 11 | 9 | 9 | 7 |
| 412 | 10 | 19 | 9 | 11 | 7 | 11 | 9 | 9 | 7 |
| 410 | 10 | 19 | 9 | 11 | 7 | 11 | 9 | 9 | 7 |
| 408 | 10 | 19 | 9 | 11 | 7 | 11 | 9 | 9 | 7 |
| 406 | 10 | 19 | 9 | 11 | 7 | 11 | 9 | 9 | 7 |
| 404 | 10 | 19 | 9 | 11 | 7 | 11 | 9 | 9 | 7 |
| 402 | 10 | 19 | 9 | 11 | 7 | 11 | 9 | 9 | 7 |
| 400 | 10 | 19 | 9 | 11 | 7 | 11 | 9 | 9 | 7 |
| 398 | 10 | 19 | 9 | 11 | 7 | 11 | 9 | 9 | 7 |
| 396 | 10 | 19 | 9 | 11 | 7 | 11 | 9 | 9 | 7 |
| 394 | 10 | 19 | 9 | 11 | 7 | 11 | 9 | 9 | 7 |
| 392 | 10 | 19 | 9 | 11 | 7 | 11 | 9 | 9 | 7 |
| 390 | 10 | 19 | 9 | 11 | 7 | 11 | 9 | 9 | 7 |
| 388 | 10 | 19 | 9 | 11 | 7 | 11 | 9 | 9 | 7 |
| 386 | 10 | 19 | 9 | 11 | 7 | 11 | 9 | 9 | 7 |
| 384 | 10 | 19 | 9 | 11 | 7 | 11 | 9 | 9 | 7 |
| 382 | 10 | 19 | 9 | 11 | 7 | 11 | 9 | 9 | 7 |
| 380 | 10 | 19 | 9 | 11 | 7 | 11 | 9 | 9 | 7 |
| 378 | 10 | 19 | 9 | 11 | 7 | 11 | 9 | 9 | 7 |
| 376 | 10 | 19 | 9 | 11 | 7 | 11 | 9 | 9 | 7 |
| 374 | 10 | 19 | 9 | 11 | 7 | 11 | 9 | 9 | 7 |
| 372 | 10 | 19 | 9 | 11 | 7 | 11 | 9 | 9 | 7 |
| 370 | 10 | 19 | 9 | 11 | 7 | 11 | 9 | 9 | 7 |
| 368 | 10 | 19 | 9 | 11 | 7 | 11 | 9 | 9 | 7 |
| 366 | 10 | 19 | 9 | 11 | 7 | 11 | 9 | 9 | 7 |
| 364 | 10 | 19 | 9 | 11 | 7 | 11 | 9 | 9 | 7 |
| 362 | 10 | 19 | 9 | 11 | 7 | 11 | 9 | 9 | 7 |
| 360 | 10 | 19 | 9 | 11 | 7 | 11 | 9 | 9 | 7 |
| 358 | 10 | 19 | 9 | 11 | 7 | 11 | 9 | 9 | 7 |
| 356 | 10 | 19 | 9 | 11 | 7 | 11 | 9 | 9 | 7 |
| 354 | 10 | 19 | 9 | 11 | 7 | 11 | 9 | 9 | 7 |
| 352 | 10 | 19 | 9 | 11 | 7 | 11 | 9 | 9 | 7 |
| 350 | 10 | 19 | 9 | 11 | 7 | 11 | 9 | 9 | 7 |
| 348 | 10 | 19 | 9 | 11 | 7 | 11 | 9 | 9 | 7 |
| 346 | 10 | 19 | 9 | 11 | 7 | 11 | 9 | 9 | 7 |
| 344 | 10 | 19 | 9 | 11 | 7 | 11 | 9 | 9 | 7 |
| 342 | 10 | 19 | 9 | 11 | 7 | 11 | 9 | 9 | 7 |
| 340 | 10 | 19 | 9 | 11 | 7 | 11 | 9 | 9 | 7 |
| 338 | 10 | 19 | 9 | 11 | 7 | 11 | 9 | 9 | 7 |
| 336 | 10 | 19 | 9 | 11 | 7 | 11 | 9 | 9 | 7 |
| 334 | 10 | 19 | 9 | 11 | 7 | 11 | 9 | 9 | 7 |
| 332 | 10 | 19 | 9 | 11 | 7 | 11 | 9 | 9 | 7 |
| 330 | 10 | 19 | 9 | 11 | 7 | 11 | 9 | 9 | 7 |
| 328 | 10 | 19 | 9 | 11 | 7 | 11 | 9 | 9 | 7 |
| 326 | 10 | 19 | 9 | 11 | 7 | 11 | 9 | 9 | 7 |
| 324 | 10 | 19 | 9 | 11 | 7 | 11 | 9 | 9 | 7 |
| 322 | 10 | 19 | 9 | 11 | 7 | 11 | 9 | 9 | 7 |
| 320 | 10 | 19 | 9 | 11 | 7 | 11 | 9 | 9 | 7 |
| 318 | 10 | 19 | 9 | 11 | 7 | 11 | 9 | 9 | 7 |
| 316 | 10 | 19 | 9 | 11 | 7 | 11 | 9 | 9 | 7 |
| 314 | 10 | 19 | 9 | 11 | 7 | 11 | 9 | 9 | 7 |
| 312 | 10 | 19 | 9 | 11 | 7 | 11 | 9 | 9 | 7 |
| 310 | 10 | 19 | 9 | 11 | 7 | 11 | 9 | 9 | 7 |
| 308 | 10 | 19 | 9 | 11 | 7 | 11 | 9 | 9 | 7 |
| 306 | 10 | 19 | 9 | 11 | 7 | 11 | 9 | 9 | 7 |
| 304 | 10 | 19 | 9 | 11 | 7 | 11 | 9 | 9 | 7 |
| 302 | 10 | 19 | 9 | 11 | 7 | 11 | 9 | 9 | 7 |
| 300 | 10 | 19 | 9 | 11 | 7 | 11 | 9 | 9 | 7 |
| 298 | 10 | 19 | 9 | 11 | 7 | 11 | 9 | 9 | 7 |
| 296 | 10 | 19 | 9 | 11 | 7 | 11 | 9 | 9 | 7 |
| 294 | 10 | 19 | 9 | 11 | 7 | 11 | 9 | 9 | 7 |
| 292 | 10 | 19 | 9 | 11 | 7 | 11 | 9 | 9 | 7 |
| 290 | 10 | 19 | 9 | 11 | 7 | 11 | 9 | 9 | 7 |
