# Supplementary material for: Cystine rather than cysteine is the preferred substrate for β-elimination by cystathionine γ-lyase: implications for dietary methionine restriction
Source: GeroScience. 2023 May 23;46(4):3617–34. doi: 10.1007/s11357-023-00788-4 (PMC11229439; doi:10.1007/s11357-023-00788-4)
Supplement: Supplementary file 3 — Supplementary file3 (DOCX 89.5 KB) [file 11357_2023_788_MOESM3_ESM.docx]

**Supplementary Table 3: Changes in the absorbance of 50 μM PLP reacting with 50, 100, 150, 200, 250, 300, 350, and 400 μM cysteine as** **are shown as a function of time and as depicted in Fig 8.**

| **Mean Values at 322 nm** | | | | | | | | |
| --- | --- | --- | --- | --- | --- | --- | --- | --- |
| PLP (uM) | 50 | 50 | 50 | 50 | 50 | 50 | 50 | 50 |
| Cysteine (uM) | 50 | 100 | 150 | 200 | 250 | 300 | 350 | 400 |
| min |  |  |  |  |  |  |  |  |
| 0 | 0.130 | 0.130 | 0.130 | 0.130 | 0.130 | 0.130 | 0.130 | 0.130 |
| 0.083333 | 0.123 | 0.124 | 0.128 | 0.126 | 0.126 | 0.129 | 0.128 | 0.129 |
| 1 | 0.123 | 0.124 | 0.128 | 0.128 | 0.127 | 0.130 | 0.131 | 0.132 |
| 2 | 0.124 | 0.126 | 0.129 | 0.130 | 0.128 | 0.132 | 0.133 | 0.134 |
| 3 | 0.125 | 0.126 | 0.131 | 0.131 | 0.131 | 0.134 | 0.136 | 0.137 |
| 4 | 0.125 | 0.127 | 0.132 | 0.133 | 0.133 | 0.136 | 0.138 | 0.140 |
| 5 | 0.126 | 0.128 | 0.133 | 0.135 | 0.135 | 0.139 | 0.141 | 0.142 |
| 6 | 0.126 | 0.129 | 0.134 | 0.137 | 0.137 | 0.141 | 0.144 | 0.145 |
| 7 | 0.127 | 0.130 | 0.135 | 0.139 | 0.139 | 0.143 | 0.146 | 0.147 |
| 8 | 0.127 | 0.131 | 0.137 | 0.140 | 0.142 | 0.146 | 0.150 | 0.152 |
| 9 | 0.127 | 0.132 | 0.138 | 0.142 | 0.143 | 0.147 | 0.152 | 0.154 |
| 10 | 0.128 | 0.133 | 0.139 | 0.143 | 0.146 | 0.149 | 0.154 | 0.156 |
| 11 | 0.128 | 0.134 | 0.140 | 0.145 | 0.146 | 0.150 | 0.154 | 0.156 |
| 12 | 0.129 | 0.135 | 0.141 | 0.146 | 0.148 | 0.151 | 0.156 | 0.158 |
| 13 | 0.129 | 0.136 | 0.142 | 0.147 | 0.149 | 0.153 | 0.158 | 0.160 |
| 14 | 0.130 | 0.137 | 0.144 | 0.149 | 0.151 | 0.154 | 0.160 | 0.162 |
| 15 | 0.130 | 0.137 | 0.145 | 0.150 | 0.152 | 0.156 | 0.162 | 0.163 |
| 16 | 0.131 | 0.138 | 0.145 | 0.152 | 0.154 | 0.157 | 0.163 | 0.165 |
| 17 | 0.131 | 0.139 | 0.147 | 0.153 | 0.155 | 0.159 | 0.165 | 0.166 |
| 18 | 0.132 | 0.140 | 0.148 | 0.154 | 0.156 | 0.160 | 0.166 | 0.168 |
| 19 | 0.132 | 0.141 | 0.149 | 0.155 | 0.158 | 0.162 | 0.168 | 0.169 |
| 20 | 0.132 | 0.141 | 0.150 | 0.156 | 0.159 | 0.163 | 0.170 | 0.171 |
| 21 | 0.133 | 0.142 | 0.151 | 0.158 | 0.160 | 0.164 | 0.171 | 0.173 |
| 22 | 0.133 | 0.143 | 0.152 | 0.159 | 0.162 | 0.166 | 0.172 | 0.174 |
| 23 | 0.134 | 0.144 | 0.153 | 0.160 | 0.163 | 0.167 | 0.174 | 0.175 |
| 24 | 0.135 | 0.145 | 0.153 | 0.161 | 0.164 | 0.168 | 0.175 | 0.177 |
| 25 | 0.135 | 0.146 | 0.155 | 0.163 | 0.165 | 0.169 | 0.176 | 0.178 |
| 26 | 0.135 | 0.145 | 0.155 | 0.164 | 0.166 | 0.170 | 0.177 | 0.179 |
| 27 | 0.136 | 0.147 | 0.156 | 0.164 | 0.168 | 0.172 | 0.179 | 0.180 |
| 28 | 0.136 | 0.148 | 0.157 | 0.166 | 0.169 | 0.173 | 0.180 | 0.181 |
| 29 | 0.136 | 0.148 | 0.158 | 0.167 | 0.170 | 0.173 | 0.181 | 0.182 |
| 30 | 0.137 | 0.149 | 0.159 | 0.168 | 0.171 | 0.175 | 0.182 | 0.184 |
| 31 | 0.137 | 0.149 | 0.160 | 0.169 | 0.172 | 0.176 | 0.183 | 0.184 |
| 32 | 0.137 | 0.150 | 0.161 | 0.169 | 0.172 | 0.177 | 0.184 | 0.185 |
| 33 | 0.138 | 0.151 | 0.161 | 0.171 | 0.174 | 0.178 | 0.185 | 0.186 |
| 34 | 0.138 | 0.151 | 0.162 | 0.172 | 0.175 | 0.179 | 0.186 | 0.187 |
| 35 | 0.139 | 0.152 | 0.163 | 0.173 | 0.175 | 0.179 | 0.187 | 0.188 |
| 36 | 0.139 | 0.153 | 0.164 | 0.174 | 0.176 | 0.180 | 0.188 | 0.189 |
| 37 | 0.139 | 0.154 | 0.164 | 0.174 | 0.177 | 0.181 | 0.189 | 0.190 |
| 38 | 0.139 | 0.154 | 0.165 | 0.175 | 0.178 | 0.182 | 0.190 | 0.191 |
| 39 | 0.140 | 0.154 | 0.166 | 0.176 | 0.179 | 0.183 | 0.191 | 0.192 |
| 40 | 0.140 | 0.155 | 0.167 | 0.176 | 0.180 | 0.184 | 0.191 | 0.193 |
| 41 | 0.140 | 0.156 | 0.167 | 0.178 | 0.180 | 0.184 | 0.192 | 0.193 |
| 42 | 0.141 | 0.156 | 0.168 | 0.179 | 0.181 | 0.185 | 0.192 | 0.194 |
| 43 | 0.141 | 0.156 | 0.169 | 0.179 | 0.182 | 0.186 | 0.194 | 0.195 |
| 44 | 0.141 | 0.157 | 0.170 | 0.180 | 0.183 | 0.187 | 0.194 | 0.196 |
| 45 | 0.142 | 0.158 | 0.170 | 0.181 | 0.183 | 0.187 | 0.195 | 0.196 |
| 46 | 0.142 | 0.158 | 0.171 | 0.181 | 0.184 | 0.188 | 0.196 | 0.197 |
| 47 | 0.143 | 0.159 | 0.171 | 0.182 | 0.185 | 0.189 | 0.196 | 0.197 |
| 48 | 0.143 | 0.160 | 0.172 | 0.183 | 0.185 | 0.189 | 0.197 | 0.198 |
| 49 | 0.143 | 0.160 | 0.173 | 0.184 | 0.187 | 0.190 | 0.198 | 0.198 |
| 50 | 0.143 | 0.161 | 0.173 | 0.184 | 0.187 | 0.191 | 0.199 | 0.199 |
| 51 | 0.144 | 0.161 | 0.174 | 0.184 | 0.188 | 0.191 | 0.199 | 0.200 |
| 52 | 0.144 | 0.162 | 0.175 | 0.186 | 0.188 | 0.192 | 0.199 | 0.200 |
| 53 | 0.144 | 0.162 | 0.175 | 0.186 | 0.188 | 0.192 | 0.200 | 0.200 |
| 54 | 0.145 | 0.162 | 0.176 | 0.187 | 0.190 | 0.193 | 0.200 | 0.201 |
| 55 | 0.145 | 0.163 | 0.176 | 0.187 | 0.190 | 0.194 | 0.200 | 0.202 |
| 56 | 0.145 | 0.163 | 0.177 | 0.188 | 0.190 | 0.194 | 0.200 | 0.202 |
| 57 | 0.145 | 0.164 | 0.178 | 0.189 | 0.191 | 0.194 | 0.201 | 0.203 |
| 58 | 0.146 | 0.164 | 0.178 | 0.189 | 0.192 | 0.195 | 0.202 | 0.203 |
| 59 | 0.146 | 0.165 | 0.179 | 0.190 | 0.192 | 0.196 | 0.202 | 0.204 |
| 60 | 0.146 | 0.165 | 0.179 | 0.191 | 0.193 | 0.196 | 0.203 | 0.204 |

| **SD Values 322 nm** | | | | | | | | |
| --- | --- | --- | --- | --- | --- | --- | --- | --- |
| PLP (uM) | 50 | 50 | 50 | 50 | 50 | 50 | 50 | 50 |
| Cysteine (uM) | 50 | 100 | 150 | 200 | 250 | 300 | 350 | 400 |
| min |  |  |  |  |  |  |  |  |
| 0 | 0.000 | 0.000 | 0.000 | 0.000 | 0.000 | 0.000 | 0.000 | 0.000 |
| 0.083333 | 0.003 | 0.002 | 0.007 | 0.006 | 0.005 | 0.005 | 0.004 | 0.003 |
| 1 | 0.002 | 0.001 | 0.007 | 0.007 | 0.002 | 0.005 | 0.005 | 0.004 |
| 2 | 0.002 | 0.001 | 0.007 | 0.006 | 0.003 | 0.005 | 0.004 | 0.002 |
| 3 | 0.002 | 0.002 | 0.007 | 0.007 | 0.003 | 0.005 | 0.004 | 0.003 |
| 4 | 0.002 | 0.001 | 0.007 | 0.007 | 0.003 | 0.005 | 0.004 | 0.003 |
| 5 | 0.003 | 0.002 | 0.007 | 0.007 | 0.004 | 0.006 | 0.003 | 0.003 |
| 6 | 0.002 | 0.002 | 0.007 | 0.007 | 0.004 | 0.006 | 0.004 | 0.003 |
| 7 | 0.002 | 0.001 | 0.007 | 0.006 | 0.004 | 0.007 | 0.004 | 0.003 |
| 8 | 0.002 | 0.002 | 0.007 | 0.007 | 0.004 | 0.008 | 0.003 | 0.003 |
| 9 | 0.002 | 0.001 | 0.007 | 0.007 | 0.004 | 0.008 | 0.003 | 0.003 |
| 10 | 0.002 | 0.002 | 0.007 | 0.007 | 0.004 | 0.008 | 0.004 | 0.003 |
| 11 | 0.002 | 0.001 | 0.007 | 0.007 | 0.004 | 0.007 | 0.004 | 0.003 |
| 12 | 0.002 | 0.002 | 0.007 | 0.007 | 0.004 | 0.007 | 0.004 | 0.003 |
| 13 | 0.002 | 0.002 | 0.007 | 0.007 | 0.004 | 0.008 | 0.004 | 0.002 |
| 14 | 0.002 | 0.001 | 0.007 | 0.007 | 0.004 | 0.008 | 0.004 | 0.003 |
| 15 | 0.002 | 0.001 | 0.007 | 0.007 | 0.004 | 0.008 | 0.004 | 0.002 |
| 16 | 0.002 | 0.001 | 0.007 | 0.007 | 0.005 | 0.009 | 0.004 | 0.003 |
| 17 | 0.002 | 0.002 | 0.007 | 0.007 | 0.005 | 0.009 | 0.005 | 0.003 |
| 18 | 0.002 | 0.001 | 0.007 | 0.007 | 0.005 | 0.009 | 0.005 | 0.003 |
| 19 | 0.002 | 0.001 | 0.007 | 0.007 | 0.005 | 0.009 | 0.004 | 0.003 |
| 20 | 0.002 | 0.002 | 0.007 | 0.007 | 0.005 | 0.009 | 0.004 | 0.003 |
| 21 | 0.002 | 0.002 | 0.007 | 0.007 | 0.006 | 0.010 | 0.004 | 0.003 |
| 22 | 0.002 | 0.002 | 0.007 | 0.007 | 0.006 | 0.010 | 0.005 | 0.003 |
| 23 | 0.002 | 0.002 | 0.007 | 0.007 | 0.006 | 0.010 | 0.005 | 0.003 |
| 24 | 0.002 | 0.001 | 0.007 | 0.007 | 0.006 | 0.010 | 0.005 | 0.003 |
| 25 | 0.002 | 0.002 | 0.008 | 0.007 | 0.006 | 0.010 | 0.005 | 0.003 |
| 26 | 0.002 | 0.002 | 0.007 | 0.007 | 0.005 | 0.011 | 0.004 | 0.003 |
| 27 | 0.001 | 0.002 | 0.008 | 0.007 | 0.006 | 0.011 | 0.005 | 0.003 |
| 28 | 0.001 | 0.002 | 0.008 | 0.007 | 0.006 | 0.011 | 0.005 | 0.003 |
| 29 | 0.002 | 0.002 | 0.007 | 0.007 | 0.006 | 0.011 | 0.005 | 0.003 |
| 30 | 0.002 | 0.001 | 0.008 | 0.007 | 0.006 | 0.011 | 0.004 | 0.003 |
| 31 | 0.002 | 0.001 | 0.007 | 0.006 | 0.007 | 0.011 | 0.005 | 0.003 |
| 32 | 0.002 | 0.002 | 0.008 | 0.007 | 0.006 | 0.011 | 0.005 | 0.003 |
| 33 | 0.002 | 0.002 | 0.008 | 0.007 | 0.006 | 0.011 | 0.004 | 0.003 |
| 34 | 0.002 | 0.002 | 0.008 | 0.007 | 0.006 | 0.011 | 0.005 | 0.003 |
| 35 | 0.002 | 0.001 | 0.008 | 0.007 | 0.006 | 0.011 | 0.005 | 0.003 |
| 36 | 0.002 | 0.002 | 0.007 | 0.007 | 0.006 | 0.011 | 0.005 | 0.003 |
| 37 | 0.002 | 0.002 | 0.008 | 0.007 | 0.007 | 0.011 | 0.005 | 0.003 |
| 38 | 0.001 | 0.002 | 0.008 | 0.007 | 0.007 | 0.012 | 0.005 | 0.003 |
| 39 | 0.002 | 0.001 | 0.007 | 0.007 | 0.006 | 0.012 | 0.005 | 0.003 |
| 40 | 0.001 | 0.002 | 0.008 | 0.007 | 0.007 | 0.012 | 0.005 | 0.003 |
| 41 | 0.001 | 0.002 | 0.008 | 0.007 | 0.007 | 0.012 | 0.005 | 0.003 |
| 42 | 0.002 | 0.002 | 0.008 | 0.007 | 0.007 | 0.012 | 0.005 | 0.003 |
| 43 | 0.002 | 0.002 | 0.008 | 0.007 | 0.007 | 0.012 | 0.005 | 0.003 |
| 44 | 0.002 | 0.002 | 0.008 | 0.007 | 0.007 | 0.012 | 0.005 | 0.003 |
| 45 | 0.002 | 0.002 | 0.008 | 0.007 | 0.007 | 0.012 | 0.005 | 0.003 |
| 46 | 0.002 | 0.002 | 0.008 | 0.006 | 0.007 | 0.012 | 0.005 | 0.003 |
| 47 | 0.002 | 0.002 | 0.008 | 0.006 | 0.007 | 0.012 | 0.005 | 0.003 |
| 48 | 0.002 | 0.002 | 0.008 | 0.007 | 0.007 | 0.012 | 0.005 | 0.003 |
| 49 | 0.002 | 0.002 | 0.008 | 0.007 | 0.007 | 0.012 | 0.005 | 0.003 |
| 50 | 0.002 | 0.002 | 0.008 | 0.006 | 0.007 | 0.012 | 0.005 | 0.003 |
| 51 | 0.002 | 0.002 | 0.008 | 0.007 | 0.007 | 0.012 | 0.005 | 0.003 |
| 52 | 0.002 | 0.002 | 0.008 | 0.007 | 0.007 | 0.012 | 0.005 | 0.002 |
| 53 | 0.002 | 0.001 | 0.008 | 0.006 | 0.007 | 0.013 | 0.005 | 0.003 |
| 54 | 0.002 | 0.001 | 0.008 | 0.007 | 0.007 | 0.013 | 0.005 | 0.003 |
| 55 | 0.002 | 0.002 | 0.008 | 0.007 | 0.007 | 0.012 | 0.004 | 0.003 |
| 56 | 0.002 | 0.002 | 0.009 | 0.007 | 0.007 | 0.012 | 0.004 | 0.003 |
| 57 | 0.002 | 0.002 | 0.009 | 0.006 | 0.007 | 0.012 | 0.004 | 0.003 |
| 58 | 0.002 | 0.002 | 0.009 | 0.006 | 0.007 | 0.013 | 0.004 | 0.004 |
| 59 | 0.002 | 0.002 | 0.008 | 0.006 | 0.007 | 0.012 | 0.004 | 0.003 |
| 60 | 0.003 | 0.003 | 0.008 | 0.007 | 0.007 | 0.013 | 0.004 | 0.003 |

| **Mean Values 388 nm** | | | | | | | | |
| --- | --- | --- | --- | --- | --- | --- | --- | --- |
| PLP (uM) | 50 | 50 | 50 | 50 | 50 | 50 | 50 | 50 |
| Cysteine (uM) | 50 | 100 | 150 | 200 | 250 | 300 | 350 | 400 |
| min |  |  |  |  |  |  |  |  |
| 0 | 0.223 | 0.223 | 0.223 | 0.223 | 0.223 | 0.223 | 0.223 | 0.223 |
| 0.083333 | 0.219 | 0.212 | 0.199 | 0.205 | 0.180 | 0.187 | 0.175 | 0.173 |
| 1 | 0.217 | 0.209 | 0.193 | 0.201 | 0.174 | 0.176 | 0.169 | 0.166 |
| 2 | 0.216 | 0.206 | 0.191 | 0.198 | 0.169 | 0.172 | 0.163 | 0.160 |
| 3 | 0.215 | 0.204 | 0.188 | 0.195 | 0.165 | 0.168 | 0.159 | 0.155 |
| 4 | 0.215 | 0.202 | 0.186 | 0.191 | 0.162 | 0.164 | 0.154 | 0.151 |
| 5 | 0.214 | 0.201 | 0.184 | 0.189 | 0.159 | 0.159 | 0.150 | 0.146 |
| 6 | 0.213 | 0.199 | 0.181 | 0.186 | 0.156 | 0.155 | 0.146 | 0.142 |
| 7 | 0.212 | 0.197 | 0.179 | 0.183 | 0.152 | 0.152 | 0.142 | 0.138 |
| 8 | 0.211 | 0.195 | 0.177 | 0.180 | 0.149 | 0.148 | 0.138 | 0.134 |
| 9 | 0.210 | 0.194 | 0.175 | 0.178 | 0.146 | 0.144 | 0.134 | 0.130 |
| 10 | 0.209 | 0.192 | 0.172 | 0.174 | 0.143 | 0.141 | 0.130 | 0.126 |
| 11 | 0.208 | 0.190 | 0.170 | 0.172 | 0.140 | 0.137 | 0.126 | 0.123 |
| 12 | 0.207 | 0.189 | 0.168 | 0.169 | 0.137 | 0.134 | 0.123 | 0.119 |
| 13 | 0.206 | 0.187 | 0.166 | 0.167 | 0.134 | 0.131 | 0.119 | 0.116 |
| 14 | 0.205 | 0.186 | 0.164 | 0.164 | 0.131 | 0.128 | 0.116 | 0.112 |
| 15 | 0.205 | 0.185 | 0.162 | 0.162 | 0.129 | 0.125 | 0.113 | 0.109 |
| 16 | 0.203 | 0.183 | 0.160 | 0.160 | 0.126 | 0.122 | 0.110 | 0.106 |
| 17 | 0.203 | 0.181 | 0.159 | 0.157 | 0.124 | 0.120 | 0.107 | 0.103 |
| 18 | 0.202 | 0.180 | 0.157 | 0.155 | 0.121 | 0.117 | 0.104 | 0.100 |
| 19 | 0.201 | 0.178 | 0.155 | 0.153 | 0.119 | 0.114 | 0.101 | 0.098 |
| 20 | 0.201 | 0.177 | 0.153 | 0.150 | 0.116 | 0.112 | 0.099 | 0.095 |
| 21 | 0.200 | 0.175 | 0.151 | 0.148 | 0.114 | 0.109 | 0.096 | 0.092 |
| 22 | 0.199 | 0.174 | 0.150 | 0.146 | 0.112 | 0.107 | 0.093 | 0.090 |
| 23 | 0.198 | 0.173 | 0.148 | 0.144 | 0.110 | 0.105 | 0.092 | 0.087 |
| 24 | 0.197 | 0.171 | 0.147 | 0.142 | 0.108 | 0.102 | 0.089 | 0.085 |
| 25 | 0.197 | 0.170 | 0.145 | 0.140 | 0.106 | 0.100 | 0.087 | 0.083 |
| 26 | 0.196 | 0.168 | 0.144 | 0.138 | 0.104 | 0.098 | 0.085 | 0.081 |
| 27 | 0.195 | 0.167 | 0.142 | 0.137 | 0.102 | 0.096 | 0.083 | 0.079 |
| 28 | 0.194 | 0.166 | 0.140 | 0.135 | 0.100 | 0.094 | 0.081 | 0.077 |
| 29 | 0.194 | 0.165 | 0.139 | 0.133 | 0.098 | 0.092 | 0.079 | 0.075 |
| 30 | 0.193 | 0.164 | 0.138 | 0.131 | 0.097 | 0.090 | 0.077 | 0.073 |
| 31 | 0.192 | 0.163 | 0.136 | 0.129 | 0.095 | 0.088 | 0.075 | 0.071 |
| 32 | 0.192 | 0.161 | 0.135 | 0.127 | 0.093 | 0.086 | 0.073 | 0.069 |
| 33 | 0.191 | 0.160 | 0.134 | 0.126 | 0.092 | 0.084 | 0.071 | 0.068 |
| 34 | 0.190 | 0.159 | 0.132 | 0.124 | 0.090 | 0.083 | 0.070 | 0.066 |
| 35 | 0.190 | 0.159 | 0.131 | 0.123 | 0.088 | 0.081 | 0.068 | 0.065 |
| 36 | 0.190 | 0.157 | 0.129 | 0.121 | 0.087 | 0.079 | 0.066 | 0.063 |
| 37 | 0.189 | 0.156 | 0.128 | 0.120 | 0.085 | 0.078 | 0.065 | 0.061 |
| 38 | 0.188 | 0.155 | 0.127 | 0.118 | 0.084 | 0.077 | 0.064 | 0.060 |
| 39 | 0.188 | 0.154 | 0.126 | 0.116 | 0.082 | 0.075 | 0.062 | 0.059 |
| 40 | 0.187 | 0.153 | 0.124 | 0.115 | 0.081 | 0.074 | 0.061 | 0.057 |
| 41 | 0.186 | 0.152 | 0.123 | 0.113 | 0.080 | 0.072 | 0.059 | 0.056 |
| 42 | 0.186 | 0.151 | 0.122 | 0.113 | 0.078 | 0.071 | 0.058 | 0.055 |
| 43 | 0.185 | 0.150 | 0.121 | 0.111 | 0.077 | 0.069 | 0.057 | 0.053 |
| 44 | 0.185 | 0.149 | 0.120 | 0.109 | 0.076 | 0.068 | 0.056 | 0.052 |
| 45 | 0.184 | 0.148 | 0.119 | 0.108 | 0.075 | 0.067 | 0.055 | 0.051 |
| 46 | 0.184 | 0.146 | 0.118 | 0.107 | 0.073 | 0.066 | 0.053 | 0.050 |
| 47 | 0.183 | 0.146 | 0.116 | 0.106 | 0.072 | 0.064 | 0.052 | 0.049 |
| 48 | 0.182 | 0.146 | 0.115 | 0.104 | 0.071 | 0.063 | 0.051 | 0.048 |
| 49 | 0.182 | 0.144 | 0.115 | 0.103 | 0.070 | 0.062 | 0.050 | 0.047 |
| 50 | 0.182 | 0.143 | 0.114 | 0.102 | 0.069 | 0.061 | 0.049 | 0.046 |
| 51 | 0.181 | 0.142 | 0.113 | 0.101 | 0.068 | 0.060 | 0.048 | 0.045 |
| 52 | 0.181 | 0.142 | 0.112 | 0.100 | 0.067 | 0.059 | 0.047 | 0.044 |
| 53 | 0.180 | 0.141 | 0.111 | 0.098 | 0.066 | 0.057 | 0.046 | 0.043 |
| 54 | 0.179 | 0.140 | 0.110 | 0.097 | 0.065 | 0.057 | 0.045 | 0.042 |
| 55 | 0.179 | 0.139 | 0.109 | 0.096 | 0.064 | 0.056 | 0.045 | 0.041 |
| 56 | 0.178 | 0.138 | 0.108 | 0.095 | 0.063 | 0.055 | 0.044 | 0.041 |
| 57 | 0.179 | 0.137 | 0.107 | 0.094 | 0.062 | 0.054 | 0.043 | 0.040 |
| 58 | 0.178 | 0.136 | 0.106 | 0.093 | 0.061 | 0.053 | 0.042 | 0.039 |
| 59 | 0.178 | 0.136 | 0.106 | 0.093 | 0.060 | 0.052 | 0.042 | 0.038 |
| 60 | 0.177 | 0.135 | 0.105 | 0.091 | 0.059 | 0.051 | 0.041 | 0.038 |

| **SD Values 388 nm** | | | | | | | | |
| --- | --- | --- | --- | --- | --- | --- | --- | --- |
| PLP (uM) | 50 | 50 | 50 | 50 | 50 | 50 | 50 | 50 |
| Cysteine (uM) | 50 | 100 | 150 | 200 | 250 | 300 | 350 | 400 |
| min |  |  |  |  |  |  |  |  |
| 0 | 0 | 0.000 | 0.000 | 0.000 | 0.000 | 0.000 | 0.000 | 0.000 |
| 0.083333 | 0.004 | 0.004 | 0.011 | 0.006 | 0.010 | 0.007 | 0.005 | 0.003 |
| 1 | 0.004 | 0.001 | 0.011 | 0.006 | 0.008 | 0.004 | 0.005 | 0.003 |
| 2 | 0.004 | 0.000 | 0.011 | 0.005 | 0.007 | 0.004 | 0.005 | 0.002 |
| 3 | 0.004 | 0.000 | 0.011 | 0.007 | 0.007 | 0.005 | 0.004 | 0.003 |
| 4 | 0.003 | 0.000 | 0.011 | 0.006 | 0.007 | 0.004 | 0.004 | 0.003 |
| 5 | 0.004 | 0.000 | 0.010 | 0.006 | 0.007 | 0.005 | 0.004 | 0.003 |
| 6 | 0.004 | 0.001 | 0.011 | 0.006 | 0.007 | 0.004 | 0.004 | 0.003 |
| 7 | 0.004 | 0.001 | 0.011 | 0.006 | 0.007 | 0.004 | 0.004 | 0.003 |
| 8 | 0.003 | 0.000 | 0.010 | 0.006 | 0.007 | 0.005 | 0.005 | 0.003 |
| 9 | 0.003 | 0.001 | 0.010 | 0.006 | 0.007 | 0.004 | 0.005 | 0.004 |
| 10 | 0.003 | 0.000 | 0.010 | 0.006 | 0.006 | 0.004 | 0.004 | 0.003 |
| 11 | 0.003 | 0.000 | 0.010 | 0.007 | 0.006 | 0.005 | 0.005 | 0.004 |
| 12 | 0.003 | 0.000 | 0.010 | 0.007 | 0.005 | 0.004 | 0.005 | 0.004 |
| 13 | 0.002 | 0.000 | 0.010 | 0.006 | 0.005 | 0.004 | 0.005 | 0.004 |
| 14 | 0.003 | 0.001 | 0.010 | 0.006 | 0.005 | 0.004 | 0.005 | 0.004 |
| 15 | 0.003 | 0.001 | 0.010 | 0.006 | 0.005 | 0.004 | 0.005 | 0.004 |
| 16 | 0.003 | 0.001 | 0.010 | 0.006 | 0.005 | 0.004 | 0.005 | 0.004 |
| 17 | 0.003 | 0.001 | 0.010 | 0.006 | 0.005 | 0.004 | 0.006 | 0.004 |
| 18 | 0.002 | 0.001 | 0.009 | 0.006 | 0.005 | 0.004 | 0.005 | 0.004 |
| 19 | 0.003 | 0.001 | 0.009 | 0.006 | 0.005 | 0.005 | 0.006 | 0.004 |
| 20 | 0.002 | 0.001 | 0.009 | 0.006 | 0.005 | 0.004 | 0.006 | 0.004 |
| 21 | 0.002 | 0.001 | 0.009 | 0.006 | 0.005 | 0.004 | 0.006 | 0.004 |
| 22 | 0.002 | 0.001 | 0.009 | 0.006 | 0.005 | 0.005 | 0.006 | 0.004 |
| 23 | 0.002 | 0.001 | 0.009 | 0.006 | 0.005 | 0.004 | 0.006 | 0.004 |
| 24 | 0.002 | 0.002 | 0.009 | 0.007 | 0.005 | 0.004 | 0.006 | 0.005 |
| 25 | 0.002 | 0.001 | 0.009 | 0.006 | 0.005 | 0.005 | 0.006 | 0.005 |
| 26 | 0.002 | 0.001 | 0.009 | 0.007 | 0.005 | 0.005 | 0.006 | 0.005 |
| 27 | 0.002 | 0.001 | 0.009 | 0.006 | 0.005 | 0.005 | 0.006 | 0.005 |
| 28 | 0.002 | 0.002 | 0.009 | 0.006 | 0.005 | 0.004 | 0.006 | 0.004 |
| 29 | 0.002 | 0.002 | 0.009 | 0.006 | 0.004 | 0.005 | 0.006 | 0.004 |
| 30 | 0.002 | 0.002 | 0.009 | 0.006 | 0.005 | 0.005 | 0.006 | 0.004 |
| 31 | 0.002 | 0.002 | 0.009 | 0.006 | 0.005 | 0.005 | 0.006 | 0.004 |
| 32 | 0.002 | 0.002 | 0.009 | 0.006 | 0.004 | 0.005 | 0.006 | 0.004 |
| 33 | 0.002 | 0.002 | 0.009 | 0.006 | 0.005 | 0.005 | 0.006 | 0.004 |
| 34 | 0.002 | 0.002 | 0.009 | 0.006 | 0.004 | 0.005 | 0.007 | 0.004 |
| 35 | 0.002 | 0.002 | 0.009 | 0.006 | 0.004 | 0.004 | 0.007 | 0.004 |
| 36 | 0.002 | 0.001 | 0.008 | 0.006 | 0.005 | 0.004 | 0.006 | 0.004 |
| 37 | 0.002 | 0.002 | 0.008 | 0.006 | 0.004 | 0.004 | 0.006 | 0.004 |
| 38 | 0.002 | 0.002 | 0.008 | 0.006 | 0.004 | 0.004 | 0.006 | 0.004 |
| 39 | 0.002 | 0.002 | 0.008 | 0.007 | 0.004 | 0.004 | 0.006 | 0.004 |
| 40 | 0.002 | 0.001 | 0.008 | 0.006 | 0.004 | 0.004 | 0.006 | 0.004 |
| 41 | 0.002 | 0.002 | 0.008 | 0.006 | 0.004 | 0.005 | 0.007 | 0.004 |
| 42 | 0.002 | 0.001 | 0.008 | 0.006 | 0.004 | 0.005 | 0.006 | 0.004 |
| 43 | 0.002 | 0.002 | 0.008 | 0.006 | 0.004 | 0.004 | 0.006 | 0.004 |
| 44 | 0.002 | 0.002 | 0.008 | 0.006 | 0.004 | 0.004 | 0.006 | 0.004 |
| 45 | 0.002 | 0.002 | 0.008 | 0.006 | 0.004 | 0.005 | 0.006 | 0.004 |
| 46 | 0.002 | 0.002 | 0.008 | 0.006 | 0.004 | 0.004 | 0.007 | 0.004 |
| 47 | 0.002 | 0.001 | 0.008 | 0.006 | 0.004 | 0.004 | 0.006 | 0.004 |
| 48 | 0.002 | 0.002 | 0.008 | 0.006 | 0.004 | 0.004 | 0.006 | 0.004 |
| 49 | 0.002 | 0.002 | 0.008 | 0.006 | 0.004 | 0.004 | 0.006 | 0.004 |
| 50 | 0.002 | 0.002 | 0.008 | 0.006 | 0.004 | 0.004 | 0.006 | 0.004 |
| 51 | 0.002 | 0.002 | 0.008 | 0.006 | 0.004 | 0.004 | 0.006 | 0.004 |
| 52 | 0.002 | 0.002 | 0.008 | 0.006 | 0.004 | 0.004 | 0.006 | 0.004 |
| 53 | 0.002 | 0.002 | 0.008 | 0.006 | 0.004 | 0.004 | 0.006 | 0.004 |
| 54 | 0.002 | 0.002 | 0.008 | 0.006 | 0.004 | 0.004 | 0.006 | 0.004 |
| 55 | 0.002 | 0.002 | 0.008 | 0.006 | 0.004 | 0.004 | 0.007 | 0.004 |
| 56 | 0.002 | 0.003 | 0.008 | 0.006 | 0.004 | 0.004 | 0.006 | 0.004 |
| 57 | 0.002 | 0.002 | 0.008 | 0.006 | 0.004 | 0.004 | 0.006 | 0.004 |
| 58 | 0.002 | 0.002 | 0.008 | 0.006 | 0.003 | 0.004 | 0.006 | 0.004 |
| 59 | 0.002 | 0.002 | 0.007 | 0.006 | 0.004 | 0.005 | 0.006 | 0.004 |
| 60 | 0.002 | 0.002 | 0.007 | 0.006 | 0.004 | 0.004 | 0.006 | 0.003 |

| **n 322 & 388 nm** | | | | | | | | |
| --- | --- | --- | --- | --- | --- | --- | --- | --- |
| PLP (uM) | 50 | 50 | 50 | 50 | 50 | 50 | 50 | 50 |
| Cysteine (uM) | 50 | 100 | 150 | 200 | 250 | 300 | 350 | 400 |
| min |  |  |  |  |  |  |  |  |
| 0 | 5 | 4 | 12 | 7 | 12 | 10 | 9 | 7 |
| 0.083333 | 5 | 4 | 12 | 7 | 12 | 10 | 9 | 7 |
| 1 | 5 | 4 | 12 | 7 | 12 | 10 | 9 | 7 |
| 2 | 5 | 4 | 12 | 7 | 12 | 10 | 9 | 7 |
| 3 | 5 | 4 | 12 | 7 | 12 | 10 | 9 | 7 |
| 4 | 5 | 4 | 12 | 7 | 12 | 10 | 9 | 7 |
| 5 | 5 | 4 | 12 | 7 | 12 | 10 | 9 | 7 |
| 6 | 5 | 4 | 12 | 7 | 12 | 10 | 9 | 7 |
| 7 | 5 | 4 | 12 | 7 | 12 | 10 | 9 | 7 |
| 8 | 5 | 4 | 12 | 7 | 12 | 10 | 9 | 7 |
| 9 | 5 | 4 | 12 | 7 | 12 | 10 | 9 | 7 |
| 10 | 5 | 4 | 12 | 7 | 12 | 10 | 9 | 7 |
| 11 | 5 | 4 | 12 | 7 | 12 | 10 | 9 | 7 |
| 12 | 5 | 4 | 12 | 7 | 12 | 10 | 9 | 7 |
| 13 | 5 | 4 | 12 | 7 | 12 | 10 | 9 | 7 |
| 14 | 5 | 4 | 12 | 7 | 12 | 10 | 9 | 7 |
| 15 | 5 | 4 | 12 | 7 | 12 | 10 | 9 | 7 |
| 16 | 5 | 4 | 12 | 7 | 12 | 10 | 9 | 7 |
| 17 | 5 | 4 | 12 | 7 | 12 | 10 | 9 | 7 |
| 18 | 5 | 4 | 12 | 7 | 12 | 10 | 9 | 7 |
| 19 | 5 | 4 | 12 | 7 | 12 | 10 | 9 | 7 |
| 20 | 5 | 4 | 12 | 7 | 12 | 10 | 9 | 7 |
| 21 | 5 | 4 | 12 | 7 | 12 | 10 | 9 | 7 |
| 22 | 5 | 4 | 12 | 7 | 12 | 10 | 9 | 7 |
| 23 | 5 | 4 | 12 | 7 | 12 | 10 | 9 | 7 |
| 24 | 5 | 4 | 12 | 7 | 12 | 10 | 9 | 7 |
| 25 | 5 | 4 | 12 | 7 | 12 | 10 | 9 | 7 |
| 26 | 5 | 4 | 12 | 7 | 12 | 10 | 9 | 7 |
| 27 | 5 | 4 | 12 | 7 | 12 | 10 | 9 | 7 |
| 28 | 5 | 4 | 12 | 7 | 12 | 10 | 9 | 7 |
| 29 | 5 | 4 | 12 | 7 | 12 | 10 | 9 | 7 |
| 30 | 5 | 4 | 12 | 7 | 12 | 10 | 9 | 7 |
| 31 | 5 | 4 | 12 | 7 | 12 | 10 | 9 | 7 |
| 32 | 5 | 4 | 12 | 7 | 12 | 10 | 9 | 7 |
| 33 | 5 | 4 | 12 | 7 | 12 | 10 | 9 | 7 |
| 34 | 5 | 4 | 12 | 7 | 12 | 10 | 9 | 7 |
| 35 | 5 | 4 | 12 | 7 | 12 | 10 | 9 | 7 |
| 36 | 5 | 4 | 12 | 7 | 12 | 10 | 9 | 7 |
| 37 | 5 | 4 | 12 | 7 | 12 | 10 | 9 | 7 |
| 38 | 5 | 4 | 12 | 7 | 12 | 10 | 9 | 7 |
| 39 | 5 | 4 | 12 | 7 | 12 | 10 | 9 | 7 |
| 40 | 5 | 4 | 12 | 7 | 12 | 10 | 9 | 7 |
| 41 | 5 | 4 | 12 | 7 | 12 | 10 | 9 | 7 |
| 42 | 5 | 4 | 12 | 7 | 12 | 10 | 9 | 7 |
| 43 | 5 | 4 | 12 | 7 | 12 | 10 | 9 | 7 |
| 44 | 5 | 4 | 12 | 7 | 12 | 10 | 9 | 7 |
| 45 | 5 | 4 | 12 | 7 | 12 | 10 | 9 | 7 |
| 46 | 5 | 4 | 12 | 7 | 12 | 10 | 9 | 7 |
| 47 | 5 | 4 | 12 | 7 | 12 | 10 | 9 | 7 |
| 48 | 5 | 4 | 12 | 7 | 12 | 10 | 9 | 7 |
| 49 | 5 | 4 | 12 | 7 | 12 | 10 | 9 | 7 |
| 50 | 5 | 4 | 12 | 7 | 12 | 10 | 9 | 7 |
| 51 | 5 | 4 | 12 | 7 | 12 | 10 | 9 | 7 |
| 52 | 5 | 4 | 12 | 7 | 12 | 10 | 9 | 7 |
| 53 | 5 | 4 | 12 | 7 | 12 | 10 | 9 | 7 |
| 54 | 5 | 4 | 12 | 7 | 12 | 10 | 9 | 7 |
| 55 | 5 | 4 | 12 | 7 | 12 | 10 | 9 | 7 |
| 56 | 5 | 4 | 12 | 7 | 12 | 10 | 9 | 7 |
| 57 | 5 | 4 | 12 | 7 | 12 | 10 | 9 | 7 |
| 58 | 5 | 4 | 12 | 7 | 12 | 10 | 9 | 7 |
| 59 | 5 | 4 | 12 | 7 | 12 | 10 | 9 | 7 |
| 60 | 5 | 4 | 12 | 7 | 12 | 10 | 9 | 7 |
