# Supplementary figures and images for: Cystine rather than cysteine is the preferred substrate for β-elimination by cystathionine γ-lyase: implications for dietary methionine restriction
Source: GeroScience. 2023 May 23;46(4):3617–34. doi: 10.1007/s11357-023-00788-4 (PMC11229439; doi:10.1007/s11357-023-00788-4)

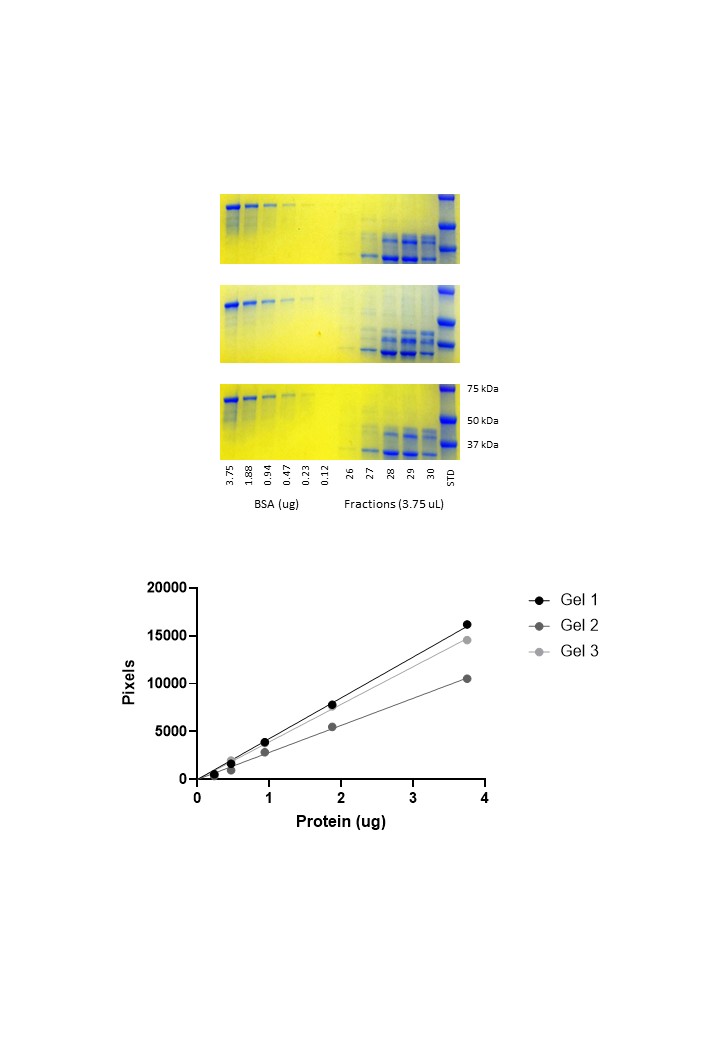

Supplement: Supplementary file 4 — Determination of the rat liver CGL protein concentrations. Chromatographic fractions of CGL and graded amounts of BSA were separated by SDS PAGE, stained with Coomassie Blue G250 and then quantified using ImageJ software. The stained gels for three separate experiments are depicted below. The protein content of the CGL fractions was estimated each gel based on the linear regression of pixels versus BSA amounts as determined using the Prism software. In each case, the R2 values for the linear regression is 1.00. (JPG 44 KB) [file 11357_2023_788_MOESM4_ESM.jpg]

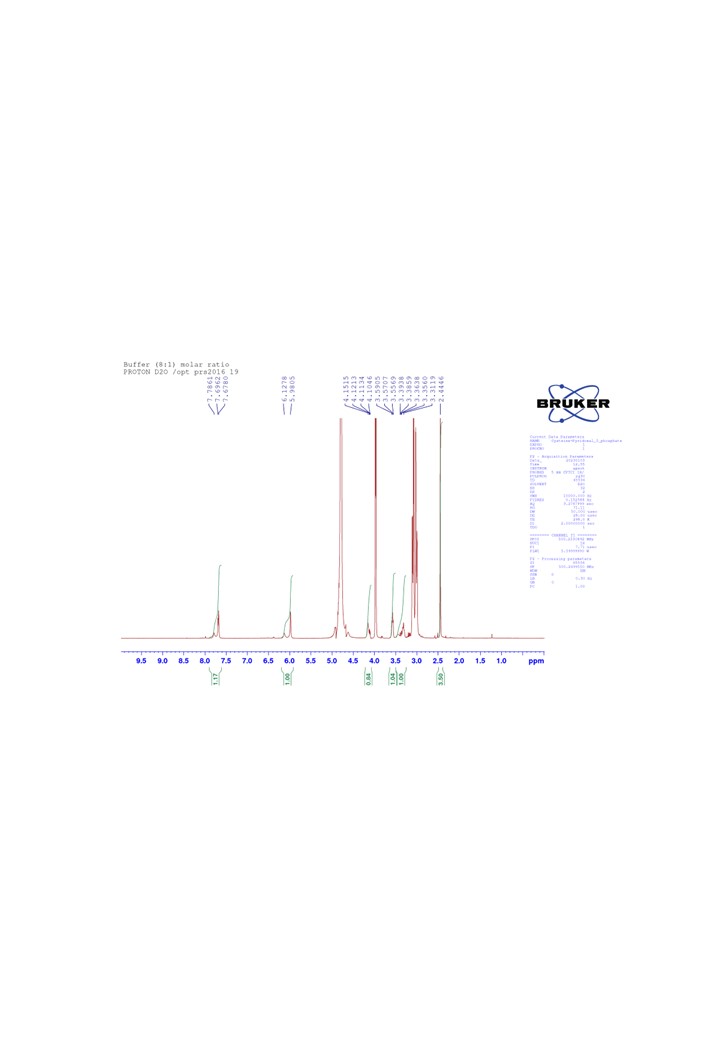

Supplement: Supplementary file 5 — Assignments of the protons in thiazolidine formed by the reaction of cysteine and PLP. (JPG 33 KB) [file 11357_2023_788_MOESM5_ESM.jpg]

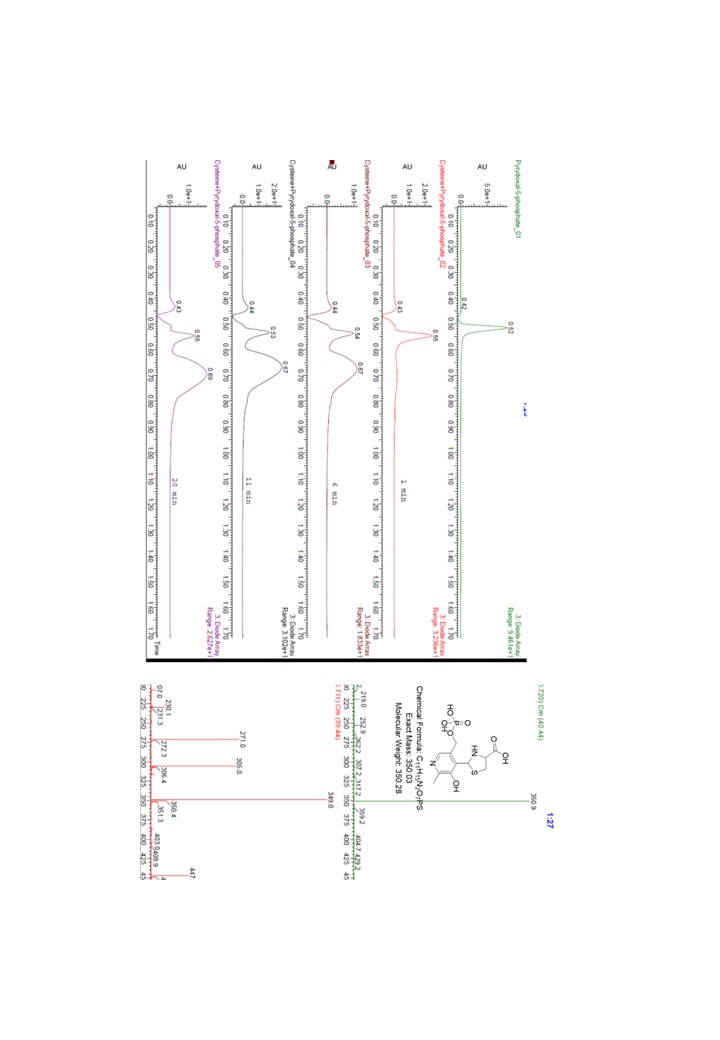

Supplement: Supplementary file 6 — Mass spectrometry of the thiazolidine formed by the reaction of cysteine and PLP. (JPG 61 KB) [file 11357_2023_788_MOESM6_ESM.jpg]

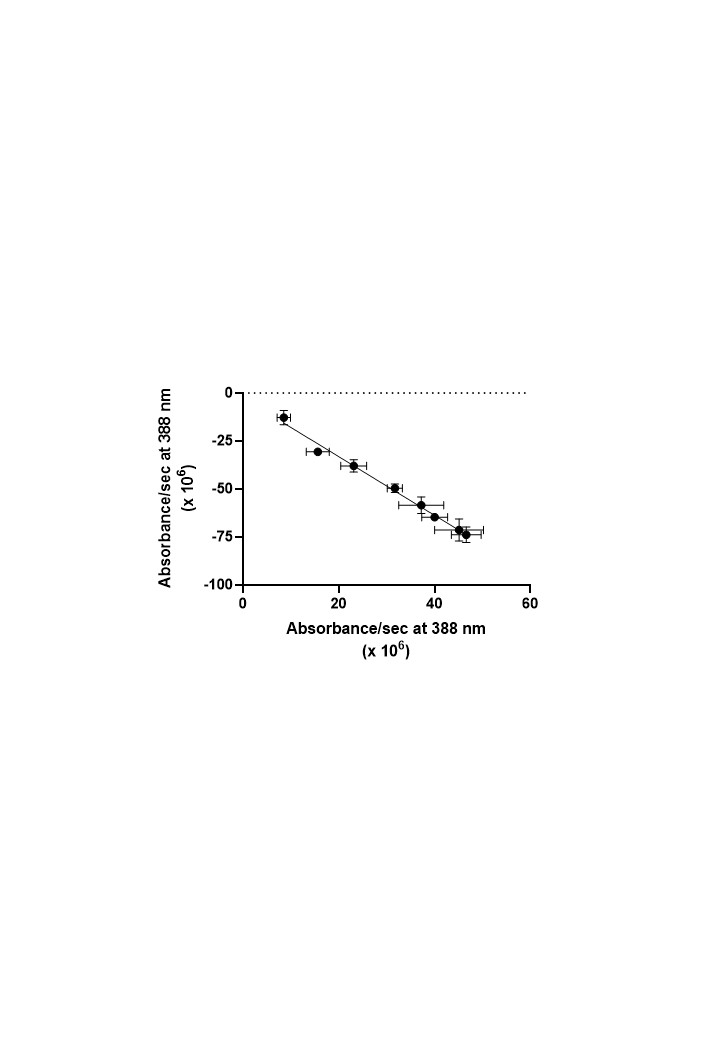

Supplement: Supplementary file 7 — Correlation between the absorbance changes at 322 and 388 nm for the reaction of PLP and cysteine. The rate changes at 388 nm are shown as a function of the rate changes at 322 nm for cysteine and PLP concentrations depicted in Figure 8. The data is taken from Figure 8B and represents mean ± SD values. (JPG 28 KB) [file 11357_2023_788_MOESM7_ESM.jpg]

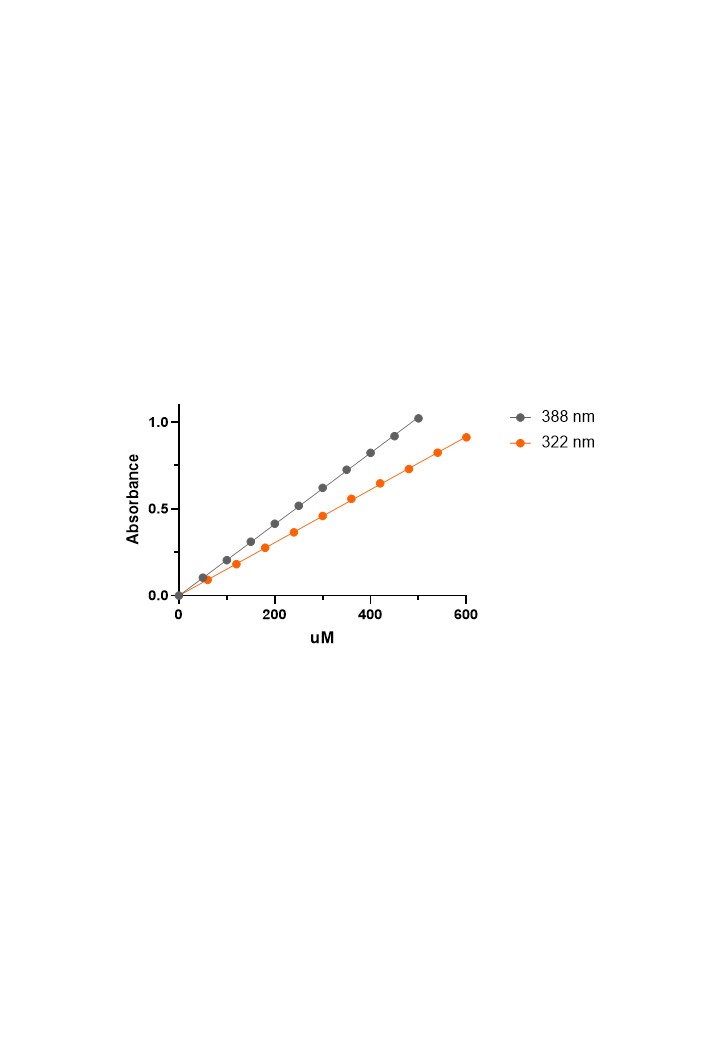

Supplement: Supplementary file 8 — Determination of the Extinction Coefficients for PLP-thiazolidine and PLP. Shown are the absorbance changes at 322 and 388 nm due to graded amounts of PLP thiazolidine and PLP, respectively. PLP thiazolidine was produced by reacting 1 mM PLP with 6 mM cystine in 100 mM potassium phosphate buffer (pH 7.2) for 15 min at 22°C, which the mixture was placed on ice. The optical densities of PLP thiazolidine concentrations at 322 nm was subtracted from the optical density for the equivalent concentrations of PLP at 322 nm. The data represents the mean values of 3 determinations that varied by less than 2% of each other. Linear regression using Prism software yielded the extinction coefficients of 1,530 and 2,060 M-1.cm-1 for the absorbance changes at 322 and 388 nm. In both cases the data was fitted to linear expressions with R2 values of 1.0. (JPG 24 KB) [file 11357_2023_788_MOESM8_ESM.jpg]

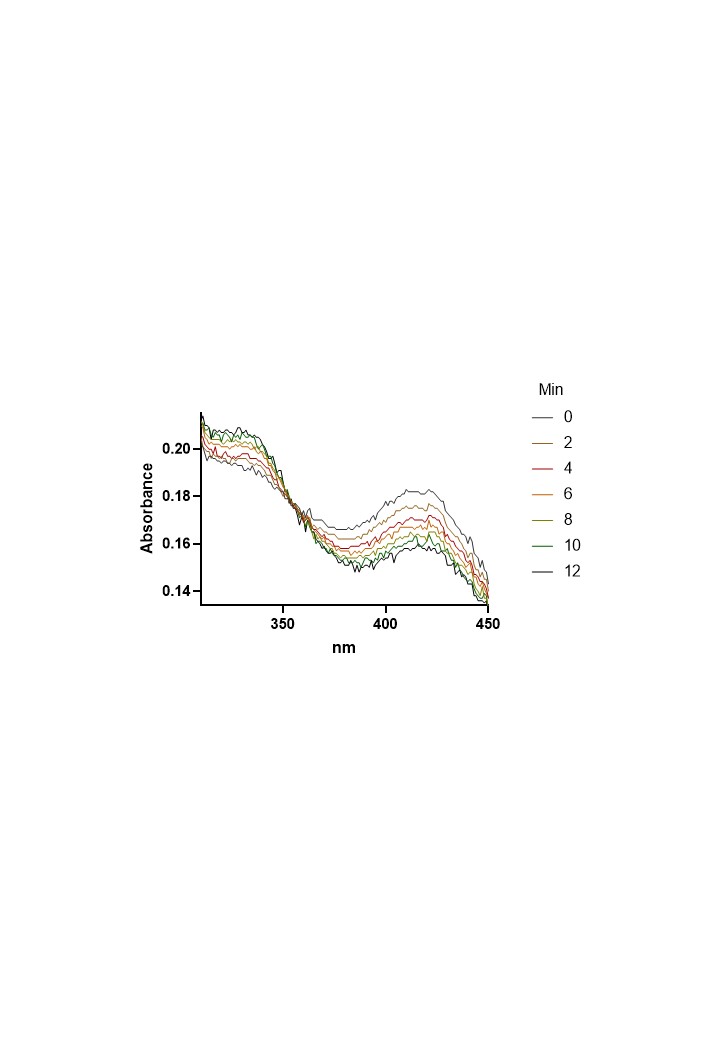

Supplement: Supplementary file 9 — Cysteine induces spectral changes in CGL indicative of a reaction with PLP. Shown are the spectra of the reaction of human recombinant CGL and cysteine at final concentrations of 5.62 and 100 μM, respectively. The reaction was carried out in 100 mM potassium phosphate buffer (pH 7.2) and at 22°C and a volume of 400 μL. Spectra were collected at a rate of 24,000 nm/min and at every min of the reaction. (JPG 32 KB) [file 11357_2023_788_MOESM9_ESM.jpg]

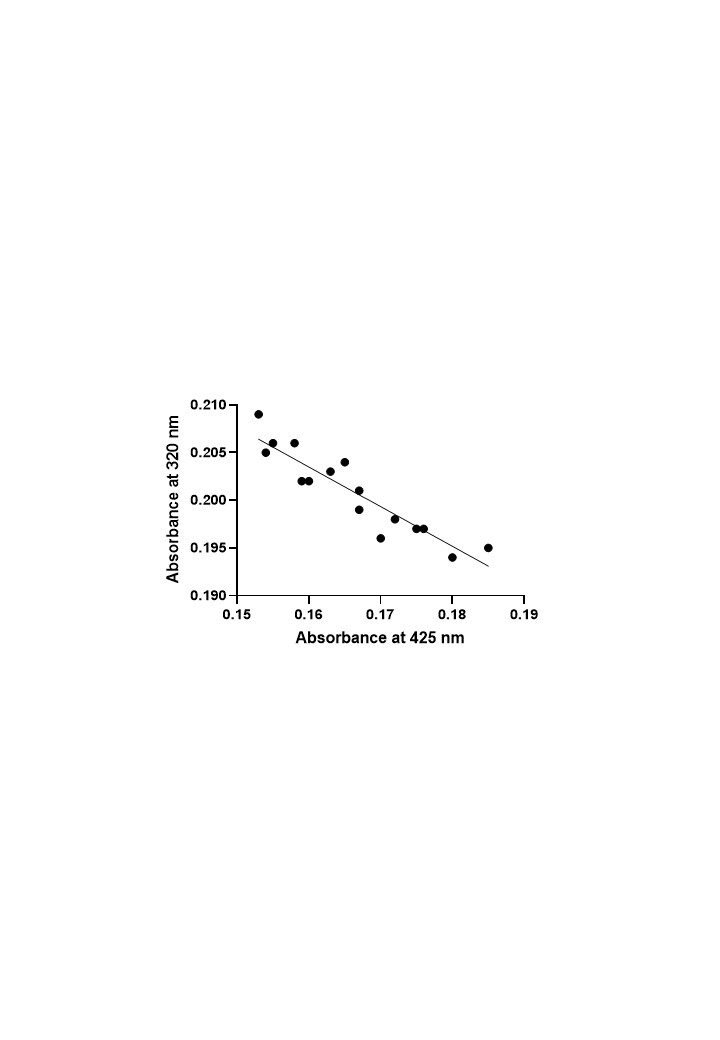

Supplement: Supplementary file 10 — Correlation between the absorbance changes at 322 and 388 nm for the reaction of CGL and cysteine The rate changes at 425 nm are shown as a function of the rate changes at 320 nm for cysteine and PLP concentrations depicted in Figure 9. (JPG 27 KB) [file 11357_2023_788_MOESM10_ESM.jpg]
